# Singular Location and Signaling Profile of Adenosine A_2A_-Cannabinoid CB_1_ Receptor Heteromers in the Dorsal Striatum

**DOI:** 10.1038/npp.2017.12

**Published:** 2017-02-15

**Authors:** Estefanía Moreno, Anna Chiarlone, Mireia Medrano, Mar Puigdellívol, Lucka Bibic, Lesley A Howell, Eva Resel, Nagore Puente, María J Casarejos, Juan Perucho, Joaquín Botta, Nuria Suelves, Francisco Ciruela, Silvia Ginés, Ismael Galve-Roperh, Vicent Casadó, Pedro Grandes, Beat Lutz, Krisztina Monory, Enric I Canela, Carmen Lluís, Peter J McCormick, Manuel Guzmán

**Affiliations:** 1 Centro de Investigación Biomédica en Red sobre Enfermedades Neurodegenerativas, Instituto de Salud Carlos III, Madrid, Spain; 2 Department of Biochemistry and Molecular Biology, University of Barcelona, Barcelona, Spain; 3 Instituto Universitario de Investigación Neuroquímica and Department of Biochemistry and Molecular Biology I, Complutense University, Madrid, Spain; 4 Instituto Ramón y Cajal de Investigación Sanitaria, Madrid, Spain; 5 School of Pharmacy, University of East Anglia, Norwich Research Park, Norwich, UK; 6 School of Biological and Chemical Sciences, Queen Mary, University of London, London, UK; 7 Department of Neurosciences, University of the Basque Country UPV/EHU, Leioa, Spain; 8 Achucarro Basque Center for Neuroscience, Bizkaia Science and Technology Park, Zamudio, Spain; 9 Biomedical Science Department, School of Medicine; Institut d’Investigacions Biomèdiques August Pi i Sunyer, and Neuroscience Institute, Barcelona University, Barcelona, Spain; 10 Pharmacology Unit, Department of Pathology and Experimental Therapeutics, IDIBELL, and Neuroscience Institute, Barcelona University, Barcelona, Spain; 11 Institute of Physiological Chemistry, University Medical Center of the Johannes Gutenberg University Mainz, Mainz, Germany; 12 Faculty of Health and Medical Sciences, University of Surrey, Guildford, Surrey, UK

## Abstract

The dorsal striatum is a key node for many neurobiological processes such as motor activity, cognitive functions, and affective processes. The proper functioning of striatal neurons relies critically on metabotropic receptors. Specifically, the main adenosine and endocannabinoid receptors present in the striatum, ie, adenosine A_2A_ receptor (A_2A_R) and cannabinoid CB_1_ receptor (CB_1_R), are of pivotal importance in the control of neuronal excitability. Facilitatory and inhibitory functional interactions between striatal A_2A_R and CB_1_R have been reported, and evidence supports that this cross-talk may rely, at least in part, on the formation of A_2A_R-CB_1_R heteromeric complexes. However, the specific location and properties of these heteromers have remained largely unknown. Here, by using techniques that allowed a precise visualization of the heteromers *in situ* in combination with sophisticated genetically modified animal models, together with biochemical and pharmacological approaches, we provide a high-resolution expression map and a detailed functional characterization of A_2A_R-CB_1_R heteromers in the dorsal striatum. Specifically, our data unveil that the A_2A_R-CB_1_R heteromer (i) is essentially absent from corticostriatal projections and striatonigral neurons, and, instead, is largely present in striatopallidal neurons, (ii) displays a striking G protein-coupled signaling profile, where co-stimulation of both receptors leads to strongly reduced downstream signaling, and (iii) undergoes an unprecedented dysfunction in Huntington’s disease, an archetypal disease that affects striatal neurons. Altogether, our findings may open a new conceptual framework to understand the role of coordinated adenosine-endocannabinoid signaling in the indirect striatal pathway, which may be relevant in motor function and neurodegenerative diseases.

## INTRODUCTION

The dorsal striatum is a key node for many neurobiological processes such as motor activity, cognitive functions, and affective processes. The vast majority (~95%) of neurons within the striatum are GABAergic medium spiny neurons (MSNs), which receive glutamatergic inputs primarily from the cortex. MSNs differ in their neurochemical composition and form two major efferent pathways, the direct (striatonigral) pathway and the indirect (striatopallidal) pathway (Kreitzer, 2009). The proper functioning of MSNs relies critically on metabotropic receptor signaling. Many neurotransmitters and neuromodulators such as dopamine, glutamate, endocannabinoids and adenosine control MSN activity and plasticity by engaging their cognate G protein-coupled receptors (GPCRs) (Lovinger, 2010; Girault, 2012). Specifically, the main endocannabinoid and adenosine receptors present in MSNs, ie, cannabinoid type 1 receptor (CB_1_R) and adenosine subtype 2A receptor (A_2A_R), are of pivotal importance in the control of neuronal excitability. CB_1_R is one of the most abundant GPCRs in MSNs (Glass *et al*, 2000; Castillo *et al*, 2012). In particular, CB_1_R is highly expressed in the terminals of both striatonigral and striatopallidal MSNs, where it mediates endocannabinoid-dependent inhibition of GABA release, thus decreasing motor activity (Katona and Freund, 2008; Castillo *et al*, 2012). CB_1_R is also expressed in glutamatergic terminals projecting from the cortex onto the striatum, where it controls MSN function by blunting glutamatergic output and mediating the so-called endocannabinoid-dependent long-term depression (Kreitzer, 2009; Castillo *et al*, 2012). A_2A_R is also very abundant in the striatum (Schiffmann and Vanderhaeghen, 1993; Schiffmann *et al*, 2007). Presynaptically, a significant fraction of the corticostriatal projections that expresses CB_1_R also contains A_2A_R. These A_2A_R molecules are mostly located on corticostriatal terminals that form synaptic contacts with striatonigral MSNs (Quiroz *et al*, 2009; Ferreira *et al*, 2015). Blockade of presynaptic A_2A_R counteracts glutamate release and motor output evoked by cortical stimulation (Quiroz *et al*, 2009; Orru *et al*, 2011; Tebano *et al*, 2012). Postsynaptically, A_2A_R is selectively located on striatopallidal MSNs, which co-express dopamine D_2_ receptor (D_2_R) (Schiffmann *et al*, 2007; Azdad *et al*, 2009; Tebano *et al*, 2012). Blockade of postsynaptic A_2A_R mediates the motor-activating effects of A_2A_R antagonists, consistent with an inactivation of the indirect pathway (Orru *et al*, 2011; Tebano *et al*, 2012).

The high expression of A_2A_R and CB_1_R in the striatum, together with the key involvement of both receptors in the control of motor and goal-directed behaviors, have led to a large number of studies on the interactions between them (Ferre *et al*, 2010; Tebano *et al*, 2012). Understanding these interactions is of special relevance not only physiologically but also pharmacologically as these receptors are targets of widely consumed psychoactive substances such as caffeine (an A_2A_R antagonist) and Δ^9^-tetrahydrocannabinol (a CB_1_R agonist). Both facilitatory and inhibitory functional interactions between striatal A_2A_R and CB_1_R have been demonstrated (Ferre *et al*, 2010; Tebano *et al*, 2012; Justinova *et al*, 2014). The precise molecular mechanisms underlying the cross-talk between these receptors is yet to be fully understood, but some evidence supports that they may rely, at least in part, on the formation of A_2A_R-CB_1_R heteromeric complexes (Carriba *et al*, 2007; Ferre *et al*, 2010; Tebano *et al*, 2012; Chiodi *et al*, 2016). Despite >10 years of research on GPCR heteromers, there continues to be a major gap in our understanding of where exactly heteromers are expressed as well as linking them to precise signal transduction pathways and biological functions. In the case of the A_2A_R-CB_1_R heteromer, factors to consider include (i) the additional partners with which A_2A_R and CB_1_R could interact differently at presynaptic sites (eg, A_1_R) (Ciruela *et al*, 2006) or postsynaptic sites (eg, D_2_R and mGluR_5_) (Navarro *et al*, 2008; Azdad *et al*, 2009; Cabello *et al*, 2009; Bonaventura *et al*, 2014; Bonaventura *et al*, 2015), (ii) the convergence of adenosine and endocannabinoid actions on various intracellular signaling pathways (Ferre *et al*, 2010; Tebano *et al*, 2012), and (iii) the intricate network of molecular processes controlling adenosine and endocannabinoid release (Kreitzer and Malenka, 2005; Lerner *et al*, 2010).

Previous studies on the A_2A_R-CB_1_R heteromer have relied essentially on energy transfer-based assays in cells ectopically expressing A_2A_R and CB_1_R, as well as co-immunolocalization and co-immunoprecipitation experiments (Carriba *et al*, 2007; Navarro *et al*, 2008; Bonaventura *et al*, 2014). These approaches, although widely exploited and certainly valuable, possess limitations of spatial resolution (co-immunolocalization), molecular specificity (co-immunoprecipitation), and biological interpretation (energy transfer using protein overexpression) to characterize GPCR heteromers. Hence, here we made use of techniques to allow a precise visualization of the heteromers *in situ* in combination with sophisticated genetically modified mouse models, together with biochemical and pharmacological approaches, to cogently characterize the anatomy and signaling profile of the A_2A_R-CB_1_R heteromer in the dorsal striatum.

## MATERIALS AND METHODS

The experimental procedures used in this study are extensively described in Supplementary Materials and Methods. That section provides precise details on animal models (genetic mouse models to study the location of the A_2A_R-CB_1_R heteromer, as well as mouse models of Huntington’s disease (HD)), human *post mortem* brain samples (see also Supplementary Table S1), recombinant adeno-associated viral vectors, HIV TAT peptides designed to disrupt the A_2A_R-CB_1_R heteromer, cell culture and transfection procedures, *in situ* proximity ligation assays (PLA), fluorescence complementation assays, dynamic mass redistribution (DMR) label-free assays, cAMP and Ca^2+^ concentration assays, western blotting assays, immunomicroscopy procedures, and statistical analyses (see also Supplementary Table S2).

## RESULTS

### A_2A_R-CB_1_R Heteromers are Located on GABAergic Neurons Rather Than Glutamatergic Projections in the Mouse Dorsal Striatum

**Figure 1:**
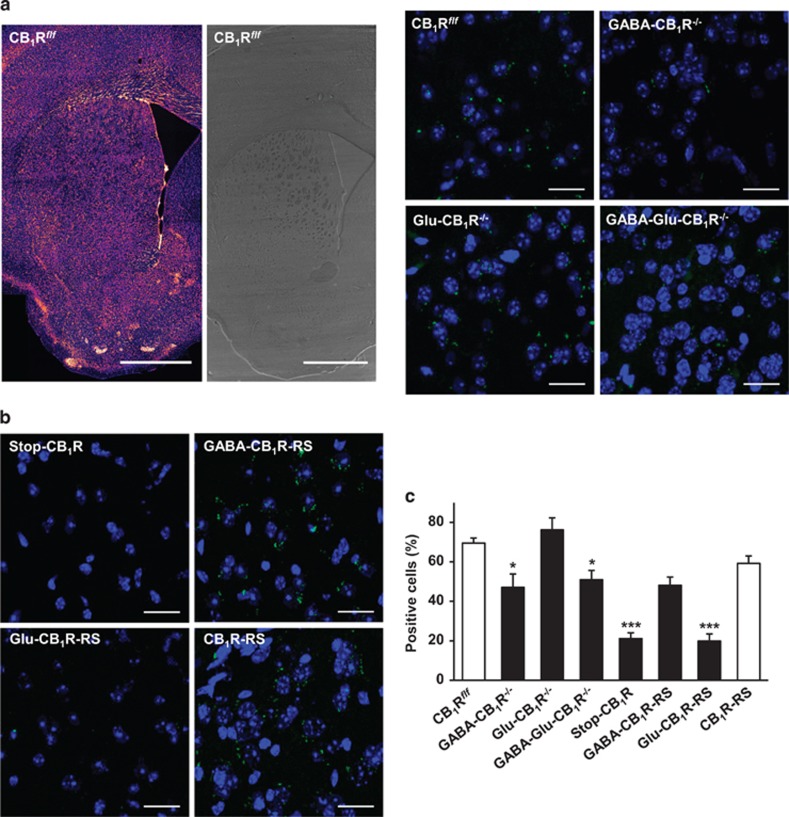
A_2A_R-CB_1_R heteromers are located on GABAergic neurons rather than glutamatergic projections in the mouse dorsal striatum. (a, b) PLA assays were performed in dorsal-striatum sections from 3–4-month-old mice of different genotypes. A_2A_R-CB_1_R heteromers are shown as green dots. Nuclei are colored in blue by DAPI staining. (a) Representative low-magnification image of tissue sections used for PLA assays. Left, DAPI-stained field; right, bright field. Scale bar: 1 mm. Representative pictures from control CB_1_R-floxed, GABA-CB_1_R^−/−^, Glu-CB_1_R^−/−^, and GABA-Glu-CB_1_R^−/−^ mice. Scale bar: 20 μm. (b) Representative pictures from Stop-CB_1_R, GABA-CB_1_R-RS mice, Glu-CB_1_R-RS mice and CB_1_R-RS mice. Scale bar: 20 μm. (c) Quantification of the number of cells containing one or more dots expressed as the percentage of the total number of cells (blue nuclei). Data are the mean±SEM of counts in 5–14 different fields from three different animals of each type. One-way ANOVA followed by Dunnet *post hoc* test showed a significant (*p<0.05, ***p<0.001) decrease of heteromer expression compared to control CB_1_R-floxed mice (a) or to CB_1_R-RS mice (b). Further details of statistical analyses are given in Supplementary Table S2.

To clarify the precise location of A_2A_R-CB_1_R heteromers in the dorsal striatum we conducted PLA experiments. The PLA assay is a powerful and straightforward technique to detect protein–protein interactions in general, and GPCR oligomers in particular, and to localize these complexes *in situ* with cell sub-population selectivity, thus allowing an unbiased demonstration and quantification of protein complexes in unmodified cells and tissues (Taura et al, 2015). Importantly, PLA permits assessing close proximity between proteins within an oligomer with high resolution (<40 nm). As PLA relies on the amplification of a small signal, its main limitation is antibody specificity/background noise, which we minimize by adapting refined technical protocols as well as employing multiple genetic mouse models and controls (Taura *et al*, 2015). Here, we first used conditional mutant mice bearing a genetic deletion of CB_1_R in forebrain GABAergic neurons (CB_1_R^*floxed/floxed;Dlx5/6-Cre/+*^ mice; herein referred to as GABA-CB_1_R^−/−^ mice) or dorsal telencephalic glutamatergic neurons (CB_1_R^*floxed/floxed;Nex-Cre/+*^ mice; herein referred to as Glu-CB_1_R^−/−^ mice) (Monory *et al*, 2006). Striatal A_2A_R-CB_1_R heteromers were evident almost exclusively as dots in the vicinity of cell nuclei, and showed a remarkable reduction in GABA-CB_1_R^−/−^ mice (Figure 1a and c). In contrast, no significant differences were observed between Glu-CB_1_R^−/−^ mice and CB_1_R^*floxed/floxed;+/+*^ controls (Figure 1a and c) when data were expressed either as a percentage of cells containing one or more dots relative to total cell nuclei (Figure 1c) or as a total number of dots relative to total cell nuclei (CB_1_R^*floxed/floxed*^ mice: 2.23±0.16; Glu-CB_1_R^−/−^ mice: 2.40±0.20; *n*=3 animals of each genotype). In addition, Glu-CB_1_R^−/−^ mice did not show any significant reduction in the percentage of A_2A_R-CB_1_R heteromer-positive cells relative to total cell nuclei in their motor cortices (CB_1_R^*floxed/floxed*^ mice: 70.3±2.3; Glu-CB_1_R^−/−^ mice: 71.4±3.0; *n*=3 animals of each genotype). Likewise, the expression levels of A_2A_R-CB_1_R heteromers displayed by GABA-CB_1_R^−/−^ mice were not decreased further when the CB_1_R gene was simultaneously ablated in glutamatergic neurons (CB_1_R^*floxed/floxed;Dlx5/6-Cre;Nex-Cre*^ mice; herein referred to as GABA-Glu-CB_1_R^−/−^ mice) (Bellocchio *et al*, 2010) (Figure 1a and c). Control experiments conducted in the absence of one of the two primary antibodies, as well as in full CB_1_R^−/−^ mice (Marsicano *et al*, 2002) and full A_2A_R^−/−^ mice (Ledent *et al*, 1997), provided strong support to the specificity of the PLA analyses performed (Supplementary Figure S1a–c). Of note, a different anti-CB_1_R primary antibody provided a similar A_2A_R-CB_1_R heteromer detection (Supplementary Figure S1d). Moreover, the specificity of the primary antibodies used was also demonstrated by immunocytofluorescence studies conducted in HEK-293T cells transfected or not with cDNAs encoding human A_2A_R or human CB_1_R (Supplementary Figure S1e).

To unequivocally ascribe A_2A_R-CB_1_R heteromers to GABAergic neurons we made use of a Cre-mediated, lineage-specific CB_1_R re-expression/rescue strategy in a CB_1_R-null background (herein referred to as Stop-CB_1_R mice) (Ruehle *et al*, 2013; De Salas-Quiroga *et al*, 2015). The selective rescue of CB_1_R expression in forebrain GABAergic neurons (herein referred to as GABA-CB_1_R-RS mice) was achieved by expressing Cre under the regulatory elements of the Dlx5/6 gene (De Salas-Quiroga *et al*, 2015). In parallel, we rescued CB_1_R expression selectively in dorsal telencephalic glutamatergic neurons (herein referred to as Glu-CB_1_R-RS mice) by using a Nex-Cre mouse line (Ruehle *et al*, 2013). As a control, an EIIa-Cre-mediated, global CB_1_R expression-rescue in a CB_1_R-null background was conducted (herein referred to as CB_1_R-RS mice) (Ruehle *et al*, 2013). Remarkably, the expression levels of A_2A_R-CB_1_R heteromers were notably restored in GABA-CB_1_R-RS mice (Figure 1b and c). In contrast, no significant rescue of the heteromer was observed in Glu-CB_1_R-RS animals when data were expressed either as a percentage of cells containing one or more dots relative to total cell nuclei (Figure 1c) or as a total number of dots relative to total cell nuclei (Stop-CB_1_R mice: 0.24±0.01; Glu-CB_1_R-RS mice: 0.28±0.04; *n*=3 animals of each genotype).

Taken together, these data strongly support that, in the mouse dorsal striatum, A_2A_R-CB_1_R heteromers are located on GABAergic neurons rather than glutamatergic projections.

### A_2A_R-CB_1_R Heteromers are Located on Indirect-Pathway MSNs in the Mouse Dorsal Striatum

The vast majority (~95%) of neurons within the striatum are MSNs (Kreitzer, 2009). These neurons differ in their neurochemical composition and form two major efferent pathways. The direct pathway consists of MSNs expressing markers such as dopamine D_1_ receptor (D_1_R) and substance P. It mainly projects to the substantia nigra pars reticulata and the internal segment of the globus pallidus. The indirect pathway is composed of MSNs expressing markers such as D_2_R and enkephalin. It mainly projects to the external segment of the globus pallidus, which, in turn, projects to the subthalamic nucleus (Kreitzer, 2009). CB_1_R is located on both direct-pathway and indirect-pathway MSNs, whereas A_2A_R resides essentially on indirect-pathway MSNs (Schiffmann *et al*, 2007; Kreitzer, 2009; Castillo *et al*, 2012). As a consequence, A_2A_R-CB_1_R heteromers would conceivably be located on indirect-pathway MSNs. To substantiate this possibility, we first used conditional mutant mice bearing a genetic deletion of CB_1_R in D_1_R-expressing neurons (CB_1_R^*floxed/floxed;Drd1a-Cre/+*^ mice; herein referred to as D_1_R-CB_1_R^−/−^ mice) (Monory *et al*, 2007). No differences were observed in the expression of A_2A_R-CB_1_R heteromers, as assessed by PLA analyses, between D_1_R-CB_1_R^−/−^ mice and control mice (Supplementary Figure S2a), thus confirming that the heteromer is not located on direct-pathway MSNs. CB_1_R is essentially a presynaptic receptor that, in MSNs, resides, mainly on terminals and collaterals (Katona and Freund, 2008; Kreitzer, 2009; Castillo *et al*, 2012). Hence, we also studied the projection sites of MSNs in CB_1_R^*floxed/floxed*^ mice. Specifically, we injected stereotactically these CB_1_R^*floxed/floxed*^ mice with a recombinant adeno-associated viral vector encoding Cre (or EGFP to gain visualization of neuronal projections) into the dorsal striatum (or the motor cortex as control). Cre expression was driven by a CaMKIIα promoter, so it was confined to MSNs (injections into the striatum) or principal neurons (injections into the cortex) (Chiarlone *et al*, 2014). Cre-mediated excision of the *loxP*-flanked CB_1_R gene in dorsal-striatum MSNs of CB_1_R^*floxed/floxed*^ mice reduced the expression of A_2A_R-CB_1_R heteromers in the globus pallidus (Supplementary Figure S2b). In contrast, inactivation of the CB_1_R gene in the motor cortices of CB_1_R^*floxed/floxed*^ mice did not affect the expression of A_2A_R-CB_1_R heteromers on corticostriatal inputs (Supplementary Figure S2c).

Collectively, these data show that, in the mouse dorsal striatum, A_2A_R-CB_1_R heteromers are primarily located on indirect-pathway MSNs.

### A_2A_R-CB_1_R Heteromers Expressed in the Mouse Dorsal Striatum are Functional

**Figure 2:**
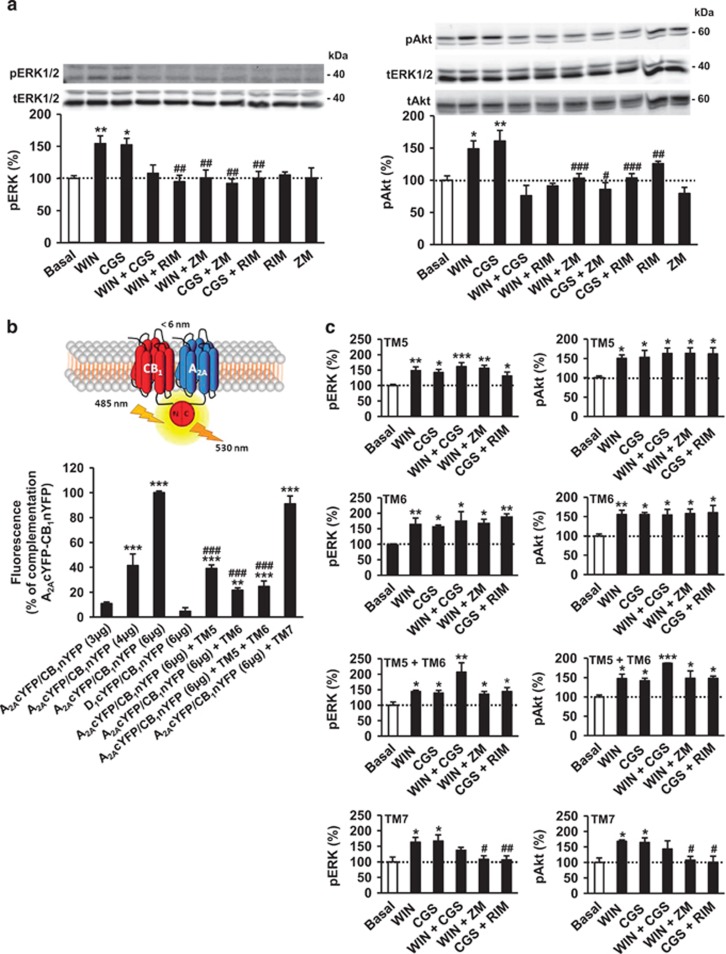
A_2A_R-CB_1_R heteromers expressed in the mouse dorsal striatum are functional. (a, c) ERK and Akt phosphorylation was determined in striatal slices from 3–4-month-old C57BL/6N mice pre-treated for 4 h with medium (a) or with 4 μM TM5, TM6 or TM7 peptides alone or in combination (c). Slices were then preincubated for 20 min with vehicle, the CB_1_R antagonist SR141716 (10 μM) or the A_2A_R antagonist ZM241385 (10 μM) before the addition of vehicle, the CB_1_R agonist WIN-55,212-2 (1 μM), the A_2A_R agonist CGS21680 (1 μM) or both, for 10 min. Immunoreactive bands from 3–6 slices from 12 different animals were quantified for each condition. Values represent mean±SEM of percentage of phosphorylation relative to basal levels found in vehicle only-treated slices (100%, dotted line). One-way ANOVA showed a significant (*p<0.05, **p<0.01, ***p<0.001) effect over basal, or of agonist plus antagonist treatment over agonist-only treatment (^#^*p*<0.05, ^##^*p*<0.01, ^###^*p*<0.001). Further details of statistical analyses are given in Supplementary Table S2. In (a), representative western blots are shown at the top of each panel. (b) Schematic representation of the bimolecular fluorescence complementation technique showing that fluorescence only appears after the YFP Venus hemiprotein (cYFP or nYFP) complementation owing to the proximity of the two receptors fused to hemi-YFP Venus proteins (top panel). In the bottom panel, fluorescence at 530 nm was monitored in HEK-293T cells transfected with the indicated amounts of cDNA encoding CB_1_R-nYFP and A_2A_R-cYFP (equal amount for each construct) or, as a negative control, transfected with cDNA encoding CB_1_R-nYFP and the non-interacting D_1_R-cYFP. Transfected cells were treated for 4 h with medium or with 4 μM TM5, TM6, and/or TM7 peptides before fluorescence reading. Values represent mean±SEM of percentage of fluorescence relative to A_2A_R-cYFP/CB_1_R-nYFP maximal complementation (*n*=4–12 replicates from three independent experiments for each condition). One-way ANOVA showed a significant change in fluorescence over non-transfected cells (**p<0.01, ***p<0.001), or of the peptide-treated over the corresponding non-peptide treated cells (^###^*p*<0.001). Further details of statistical analyses are given in Supplementary Table S2.

Previous reports have shown the existence of both facilitatory and inhibitory functional interactions between A_2A_R and CB_1_R (Ferre *et al*, 2010; Tebano *et al*, 2012). To investigate the possible role of the A_2A_R-CB_1_R heteromer in these interactions we characterized in detail heteromer functionality in the dorsal striatum. For this purpose we used C57BL/6N-mouse striatal slices and conducted cell signaling experiments on two pathways coupled to A_2A_R and CB_1_R: extracellular signal-regulated kinase (ERK) and Akt. The CB_1_R agonist WIN-55,212-2 or the A_2A_R agonist CGS21680 increased ERK phosphorylation (activation) in the dorsal striatum, whereas co-incubation with both agonists abrogated ERK phosphorylation, thus demonstrating a negative cross-talk between A_2A_R and CB_1_R (Figure 2a). In addition, the CB_1_R antagonist SR141716 (rimonabant) or the A_2A_R antagonist ZM241385 prevented the ERK-activating effect of WIN-55,212-2 or CGS21680 (Figure 2a). These data show a cross-antagonism between the two receptors, a phenomenon not uncommon in heteromers. When these cross-pharmacological assays were conducted for Akt phosphorylation (activation), similar negative cross-talk and cross-antagonism processes were observed (Figure 2a). Collectively, these findings demonstrate the existence of inhibitory interactions between A_2A_R and CB_1_R in the mouse dorsal striatum.

Next, we sought to substantiate that the aforementioned negative cross-talk and cross-antagonism between A_2A_R and CB_1_R rely on A_2A_R-CB_1_R heteromers. It is generally believed that agonist binding to the extracellular pocket of GPCRs induces local conformational changes that increase signaling by opening an intracellular cavity via the movement of transmembrane helices (TMs) 5 and 6 for receptor activation, whereas, conversely, inverse agonists decrease the basal, agonist-independent, level of signaling by closing this cavity (Shoichet and Kobilka, 2012; Venkatakrishnan *et al*, 2013). In fact, the reported crystal structure of the agonist-bound A_2A_R, compared with the inactive, antagonist-bound A_2A_R, shows an outward tilt and rotation of the cytoplasmic half of TM6 and a movement of TM5, thus resembling the changes associated with the active-state structure of other class A GPCRs (Xu *et al*, 2011). Likewise, the crystal structure of the antagonist-bound CB_1_R has been recently reported, showing a similar opsin-like behavior for this receptor (Hua *et al*, 2016; Shao *et al*, 2016). Our aforementioned observation that A_2A_R-CB_1_R heteromers display both negative cross-talk and cross-antagonism suggests a negative modulation between both receptors through protein–protein interactions involving the TM5/TM6 interface. Hence, to test this hypothesis, we studied whether synthetic peptides with the sequence of TM5, TM6 or TM7 (as negative control) of CB_1_R, fused to HIV TAT peptide to allow efficient intracellular delivery and plasma membrane insertion (Schwarze *et al*, 1999; He *et al*, 2011), were able to disrupt A_2A_R-CB_1_R heteromerization and the observed bidirectional cross-signaling. This approach has been recently used by us and others to disrupt other heteromers (Guitart *et al*, 2014; Lee *et al*, 2014; Viñals *et al*, 2015).

We first characterized the TM interference peptides by the bimolecular fluorescence complementation technique. In this assay, fluorescence only appears after correct folding of two YFP Venus hemiproteins. This occurs when two receptors fused to hemi-YFP Venus proteins (cYFP or nYFP) come within proximity to facilitate YFP Venus folding (Figure 2b, scheme). Fluorescence was detected in HEK-293T cells transfected with different amounts of cDNA encoding CB_1_R-nYFP and A_2A_R-cYFP, but not in negative controls in which cells were transfected with cDNA encoding CB_1_R-nYFP and the non-interacting D_1_R-cYFP (Figure 2b). The TM-targeted peptides were subsequently tested. We found that treatment of cells expressing CB_1_R-nYFP and A_2A_R-cYFP with TM5 or TM6 (but not TM7) peptides disrupted the heteromer structure, as revealed by a loss of fluorescence (Figure 2b). We next studied the effect of the interference peptides on A_2A_R and CB_1_R signaling in mouse striatal slices. When the peptides were evaluated in cross-pharmacological assays, we found that pretreatment of brain slices with TM5, TM6 or both (but not TM7) peptides disrupted (i) the ability of the CB_1_R agonist WIN-55,212-2 and the CB_1_R antagonist SR141716 to dampen A_2A_R-evoked actions on ERK and Akt, as well as (ii) the ability of the A_2A_R agonist CGS21680 and the A_2A_R antagonist ZM241385 to dampen CB_1_R-evoked actions on these two signaling pathways (Figure 2c). Of note, when the TM5 and TM6 peptides were used in combination, the increase in ERK and Akt phosphorylation upon receptor co-activation tended to be higher compared with TM5-only or TM6-only incubations (Figure 2c), thus conceivably reflecting that the peptide combination is more efficient than each peptide alone in disrupting the heteromer.

Together, these data provide evidence for the importance of the TM5/TM6 interface in the A_2A_R-CB_1_R heteromer, and support that the negative cross-talk and cross-antagonism that occurs between CB_1_R and A_2A_R are due to protein–protein interactions and are a specific biochemical characteristic of the A_2A_R-CB_1_R heteromer.

### Functional A_2A_R-CB_1_R Heteromers are Present in Wild-Type and Mutant Huntingtin-Expressing Striatal Neuroblasts

To evaluate the relevance of the A_2A_R-CB_1_R heteromer in a pathological setting we selected HD as a model because (i) it is the paradigmatic disease primarily caused by a selective loss of MSNs in the dorsal striatum (Walker, 2007), and (ii) changes in the expression and function of A_2A_R and CB_1_R have been shown to occur in the dorsal striatum of patients and animal models of the disease (Glass *et al*, 2000; Fernandez-Ruiz *et al*, 2011; Lee and Chern, 2014). We first characterized the heteromer in conditionally immortalized striatal neuroblasts expressing two normal (STHdh^Q7^) or mutant (STHdh^Q111^) full-length endogenous huntingtin alleles with 7 or 111 glutamine residues, respectively, which represent a widely accepted cellular model to investigate huntingtin actions. These cells do not exhibit mutant-huntingtin inclusions (Trettel *et al*, 2000), thus allowing the modeling of changes occurring at early HD stages.

**Figure 3:**
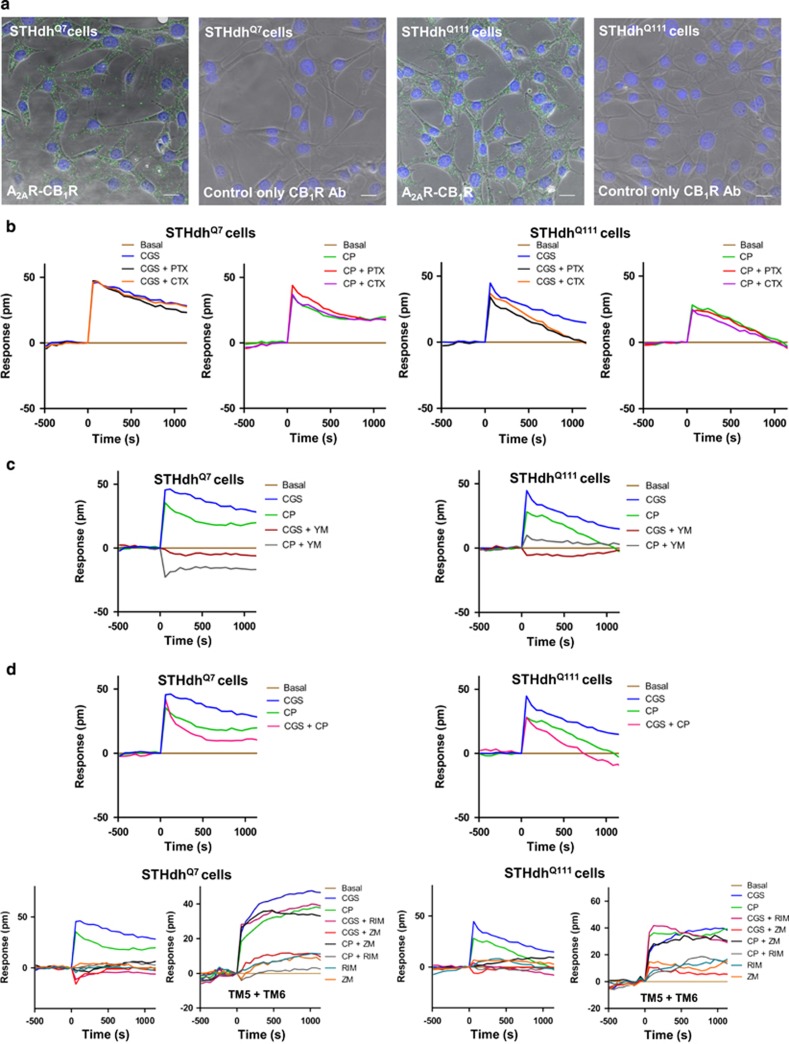
A_2A_R-CB_1_R heteromers expressed in wild-type STHdh^Q7^ and mutant huntingtin-expressing STHdh^Q111^ striatal neuroblasts signal via G_q_ protein rather than G_i_ or G_s_ protein. (a) PLA assays were performed in STHdh^Q7^ and STHdh^Q111^ cells. A_2A_R-CB_1_R heteromers are shown as green dots. Nuclei are colored in blue by DAPI staining. Controls in the absence of anti-A_2A_R primary antibody were also performed. Representative pictures are shown. Scale bar: 20 μm. (b) Dynamic mass redistribution (DMR) assays were performed in STHdh^Q7^ and STHdh^Q111^ cells pretreated overnight with vehicle, pertussis toxin (PTX; 10 ng/ml) or cholera toxin (CTX; 100 ng/ml), and further treated with vehicle, the A_2A_R agonist CGS21680 (1 μM) or the CB_1_R agonist CP-55,940 (1 μM). (c) DMR assays in STHdh^Q7^ and STHdh^Q111^ cells preincubated for 30 min with vehicle or the G_q_ protein inhibitor YM-254890 (1 μM), and then activated with the A_2A_R agonist CGS21680 (1 μM) or the CB_1_R agonist CP-55,940 (1 μM). (d) DMR assays showing negative cross-talk (top panels) and cross-antagonism (bottom panels) between A_2A_R and CB_1_R signaling. STHdh^Q7^ and STHdh^Q111^ cells were not pre-treated (top panels) or pre-treated for 4 h with medium (left bottom panels) or with 4 μM TM5 plus TM6 (right bottom panels) before incubation for 30 min with vehicle, the CB_1_R antagonist SR141716 (RIM; 1 μM) or the A_2A_R antagonist ZM241385 (1 μM), and then activated with vehicle, CGS21680 (1 μM) or CP-55,940 (1 μM). (b–d) The resulting shifts of reflected light wavelength (pm) were monitored over time. Each panel is a representative experiment of *n*=3 different experiments. Each curve is the mean of a representative optical trace experiment carried out in triplicates.

We readily detected PLA-positive A_2A_R-CB_1_R heteromers in both STHdh^Q7^ and STHdh^Q111^ cells (Figure 3a), indicating that the mere expression of mutant huntingtin does not prevent heteromerization of both receptors. To evaluate the functional characteristics of A_2A_R-CB_1_R heteromers, we first measured the global cellular response using DMR label-free assays, which detect changes in light diffraction in the bottom 150 nm of a cell monolayer. In these experiments we had a preference for CP-55,940 over WIN-55,212-2 as the CB_1_R agonist because the former is less hydrophobic than the latter and so conceivably more accessible to cultured cells. In fact, dose–response experiments conducted in both STHdh^Q7^ and STHdh^Q111^ cells showed that CP-55,940 impacted the DMR signal more markedly than WIN-55,212-2 (Supplementary Figure S3a and b). Both the A_2A_R agonist CGS21680 and the CB_1_R agonist CP-55,940 induced time-dependent signaling in STHdh^Q7^ and STHdh^Q111^ cells (Figure 3b). Of note, A_2A_R and CB_1_R-evoked signaling was essentially insensitive to pertussis toxin (PTX) or cholera toxin (CTX) (Figure 3b), thus indicating that these receptors do not significantly couple to G_i_ or G_s_ proteins in these cells. This notion was further supported by the observation that, in both STHdh^Q7^ cells (Supplementary Figure S4a) and STHdh^Q111^ cells (Supplementary Figure S4b), neither the A_2A_R agonist nor the CB_1_R agonist was able to affect basal or forskolin-elevated cAMP concentrations in the absence or presence of PTX or CTX. In line with this apparent lack of ‘classical’ A_2A_R-G_s/olf_ and CB_1_R-G_i_ coupling, the G_q_ protein inhibitor YM-254890 was able to abrogate the A_2A_R and CB_1_R-evoked changes in DMR (Figure 3c). This non-conventional coupling did appear to be due to heteromer formation as experiments conducted with the TM5 and TM6 peptides on STHdh^Q7^ and STHdh^Q111^ cells showed that the peptide combination, presumably by disrupting the heteromer, turned A_2A_R and CB_1_R action to their ‘classical’, ‘protomeric’ G_s/olf_, and G_i_-mediated signaling, respectively (Supplementary Figure S4c). This strongly supports that there is no limitation of G_s/olf_ or G_i_ protein availability in these cells, as previously indicated by others’ work (Araki *et al*, 2006), and that the A_2A_R-CB_1_R heteromer couples selectively to G_q_. Moreover, and further supporting a G_q_-dependent signaling for the heteromer, engagement of A_2A_R or CB_1_R increased intracellular free Ca^2+^ concentration in both STHdh^Q7^ and STHdh^Q111^ cells (Supplementary Figure S5).

We next investigated whether the heteromer-specific biochemical properties described above could influence G_q_-driven signaling. Regarding negative cross-talk, the DMR signal induced by the A_2A_R agonist CGS21680 alone or the CB_1_R agonist CP-55,940 alone was attenuated when both agonists were added together to STHdh^Q7^ or STHdh^Q111^ cells (Figure 3d, top panels). Regarding cross-antagonism, the DMR signal induced by the CB_1_R agonist was prevented not only by the CB_1_R antagonist SR141716 but also by the A_2A_R antagonist ZM241385, and, similarly, the DMR signal induced by the A_2A_R agonist CGS21680 was also prevented by either antagonist (Figure 3d, bottom panels). Of note, the combination of the TM5 and TM6 peptides disrupted the cross-antagonism between A_2A_R and CB_1_R in STHdh^Q7^ and STHdh^Q111^ cells (Figure 3d, bottom panels).

Collectively, these data indicate that co-expression of A_2A_R and CB_1_R, likely through the formation of A_2A_R-CB_1_R heteromers, facilitates G_q_ rather than G_s_ or G_i_ coupling in wild-type and mutant huntingtin-expressing mouse striatal neuroblasts.

### Functional A_2A_R-CB_1_R Heteromers are Expressed in HD Mice at Early but not Advanced Disease Stages

**Figure 4:**
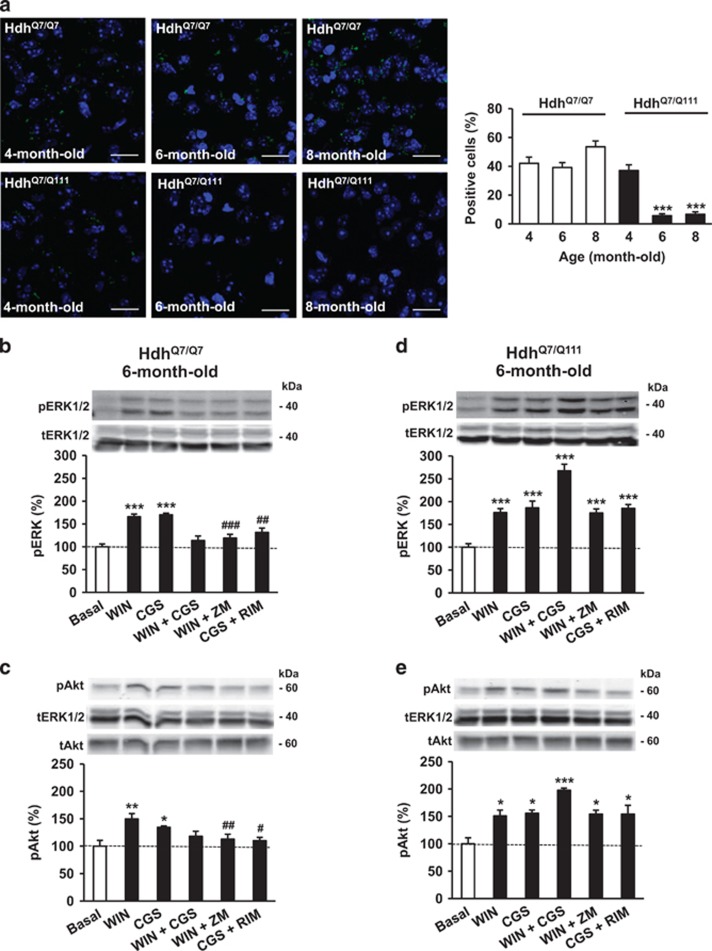
Functional A_2A_R-CB_1_R heteromers are expressed in Hdh^Q7/Q111^ HD mice at early but not advanced disease stages. (a) PLA assays were performed in dorsal-striatum sections from wild-type Hdh^Q7/Q7^ mice and mutant huntingtin-expressing knock-in Hdh^Q7/Q111^ mice. A_2A_R-CB_1_R heteromers are shown as green dots in mice at 4, 6, and 8 months of age. Nuclei are colored in blue by DAPI staining. Representative pictures are shown. Scale bar: 20 μm. Quantification of the number of cells containing one or more dots expressed as the percentage of the total number of cells (blue nuclei). Data are the mean±SEM of counts in 11–26 different fields from five different animals of each type. One-way ANOVA followed by Bonferroni *post hoc* test showed showed a significant (***p<0.001) decrease of heteromer expression in Hdh^Q7/Q111^compared with the respective age-matched Hdh^Q7/Q7^ mice. (b–e) ERK phosphorylation (b, d) and Akt phosphorylation (c, e) were determined in striatal slices from 6 month-old wild-type Hdh^Q7/Q7^ mice (b, c) and mutant huntingtin-expressing knock-in Hdh^Q7/Q111^ mice (d, e). Slices were preincubated for 20 min with vehicle, the CB_1_R antagonist SR141716 (RIM; 1 μM) or the A_2A_R antagonist ZM241385 (1 μM) before the addition of vehicle or the CB_1_R agonist WIN-55,212-2 (1 μM), the A_2A_R agonist CGS21680 (1 μM), or both, for 10 min. Immunoreactive bands from 4–6 slices of 5–6 different animals were quantified for each condition. Values represent mean±SEM of percentage of phosphorylation relative to basal levels found in vehicle only-treated slices (100%, dotted line). Representative western blots are shown at the top of each panel. One-way ANOVA showed a significant effect over basal (*p<0.05, **p<0.01, ***p<0.001), or of the antagonist plus agonist treatment over the agonist-only treatment (^#^*p*<0.05, ^##^*p*<0.01, ^###^*p*<0.001). Further details of statistical analyses are given in Supplementary Table S2.

To study the role of A_2A_R-CB_1_R heteromers in HD *in vivo* we analyzed their expression and function in a widely accepted model of HD, heterozygous mutant knock-in Hdh^Q7/Q111^ mice, that express in heterozygosity a mutant full-length huntingtin allele with 111 glutamine residues, and wild-type Hdh^Q7/Q7^ mice, that express two wild-type full-length huntingtin alleles with 7 glutamine residues. At an early stage of the disease (4 months of age), mutant Hdh^Q7/Q111^ mice displayed A_2A_R-CB_1_R heteromers in the dorsal striatum at similar levels as wild-type Hdh^Q7/Q7^ mice (Figure 4a). However, at more advanced stages (6 and 8 months of age), the expression of A_2A_R-CB_1_R heteromers was almost completely lost in mutant Hdh^Q7/Q111^ mice but not wild-type Hdh^Q7/Q7^ mice (Figure 4a). Of note, total striatal A_2A_R and CB_1_R expression, as determined by western blot (Supplementary Figure S6a) and immunofluorescence microscopy (Supplementary Figure S6b), was largely preserved in 6-month-old mutant Hdh^Q7/Q111^ mice compared with age-matched wild-type Hdh^Q7/Q7^ mice. Hence, irrespective of the small differences found between the western blot and immunofluorescence data, which can be conceivably due to the intrinsic characteristics of the two techniques, these findings suggest that the massive loss of A_2A_R-CB_1_R heteromers found in Hdh^Q7/Q111^ mice is mostly heteromer-selective and not primarily due to a mere reduction of total A_2A_R and CB_1_R molecules. In agreement with this notion, and as a further proof of the selective loss, the expression of another CB_1_R heteromer previously reported in indirect-pathway MSNs, namely CB_1_R-D_2_R (Navarro *et al*, 2008; Bonaventura *et al*, 2014), was not reduced in 6-month-old mutant Hdh^Q7/Q111^ mice compared with their wild-type controls (Supplementary Figure S6c). Moreover, a remarkable loss of A_2A_R-CB_1_R heteromers was also observed in advanced stages of mouse models of HD transgenic for human mutant huntingtin exon 1, specifically R6/1 mice (Supplementary Figure S7a) and R6/2 mice (Supplementary Figure S7b). Again, the expression of CB_1_R-D_2_R heteromers, used as a control, did not decrease in advanced-stage R6/1 or R6/2 mice compared with age-matched wild-type animals (Supplementary Figure S7c).

CB_1_R is highly abundant in most MSNs (Katona and Freund, 2008; Castillo *et al*, 2012), but it has been reported that the downregulation of CB_1_R mRNA expression in R6 transgenic mice is striatum subregion-selective, occurring preferentially in the dorsolateral than the dorsomedial striatum (Denovan-Wright and Robertson, 2000; McCaw *et al*, 2004). Hence, we analyzed the expression of total A_2A_R and CB_1_R immunoreactivity, as well as that of the A_2A_R-CB_1_R heteromer, in the dorsolateral *vs* the dorsomedial striatum of wild-type Hdh^Q7/Q7^ and mutant Hdh^Q7/Q111^ mice at 6 months of age. We found no significant differences between the two dorsal-striatum compartments in total A_2A_R immunoreactivity in either Hdh^Q7/Q7^ mice (relative values: dorsolateral: 100±5.7; A_2A_R, dorsomedial: 101.8±5.7; *n*=3 animals) or Hdh^Q7/Q111^ mice (relative values: dorsolateral: 100±5.2; A_2A_R, dorsomedial: 114.8±7.8; *n*=3 animals). There was a moderate preference of total CB_1_R protein expression for the dorsolateral striatum in Hdh^Q7/Q7^ mice (relative values: dorsolateral: 100±3.8; dorsomedial: 83.1±2.5; *n*=3 animals; *p*=0.032), as well as a non-significant trend in Hdh^Q7/Q111^ mice (relative values: dorsolateral: 100±2.9; dorsomedial: 85.8±2.7; *n*=3 animals). Regarding the A_2A_R-CB_1_R heteromer, we found no significant differences between the two dorsal-striatum compartments in the percentage of heteromer-positive cells relative to total cell nuclei in either Hdh^Q7/Q7^ mice (dorsolateral: 45.0±4.9; dorsomedial: 44.0±3.8; *n*=3 animals) or Hdh^Q7/Q111^ mice (dorsolateral: 10.4±2.3; dorsomedial: 7.5±1.4; *n*=4 animals). Overall, these data show that the A_2A_R-CB_1_R heteromer has a rather similar expression pattern in the mouse dorsolateral and dorsomedial striatum.

To study the function of the A_2A_R-CB_1_R heteromer in HD mice, we performed cross-signaling experiments in striatal slices from 6-month-old Hdh^Q7/Q7^ and Hdh^Q7/Q111^ mice. Consistently with the aforementioned data on both cell and slice cultures from control C57BL/6N mice, dual agonist treatment with WIN-55,212-2 and CGS21680 depressed phospho-ERK or phospho-Akt signal compared with single-agonist stimulation in wild-type Hdh^Q7/Q7^ mice, thus showing a negative cross-talk (Figure 4b and c). In addition, the action of both agonists was blocked when the slices were preincubated with the partner receptor antagonists, SR141716 or ZM241385, thus showing cross-antagonism (Figure 4b and c). Interestingly, in Hdh^Q7/Q111^ mice this negative cross-talk and cross-antagonism signature was not detected (Figure 4d and e), in line with the PLA data showing that the A_2A_R-CB_1_R heteromer is indeed not expressed in 6-month-old Hdh^Q7/Q111^ mice. Of note, and also in line with the data shown above, this loss of cross-signaling did not appear to be simply due to the loss of surface expression of functional receptors, as the extent of single agonist-evoked ERK and Akt stimulation was roughly equivalent in both Hdh^Q7/Q111^ and Hdh^Q7/Q7^ mice (Figure 4b–e).

Together, these data demonstrate that a selective loss of functional A_2A_R-CB_1_R heteromers accompanies disease progression in mouse models of HD.

### A_2A_R-CB_1_R Heteromers are Lost in the Caudate-Putamen of High-Grade HD Patients

**Figure 5:**
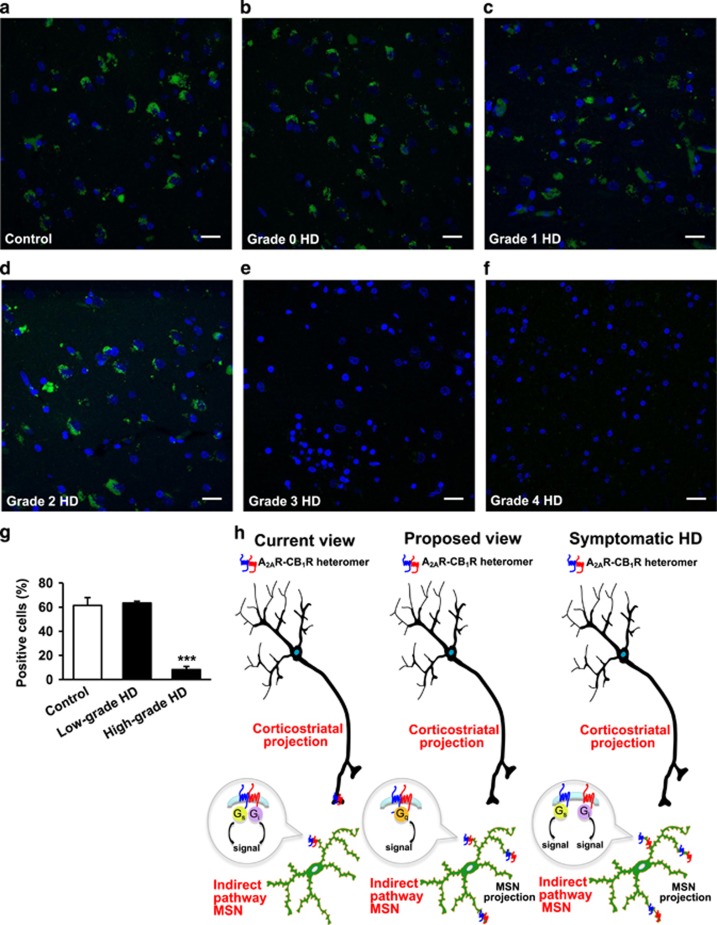
A_2A_R-CB_1_R heteromers are lost in the caudate-putamen of high-grade HD patients. PLA assays were performed in caudate-putamen sections of *post mortem* samples from control subjects (a) and HD patients at different grades (b–f). A_2A_R-CB_1_R heteromers are shown as green dots. Nuclei are colored in blue by DAPI staining. Representative pictures are shown. Scale bar: 20 μm. (g) Quantification of the number of cells containing one or more dots expressed as the percentage of the total number of cells (blue nuclei). Data are the mean±SEM of counts in 21–43 different fields from five control subjects, five low-grade HD patients (1 grade 0, 2 grade 1, plus 2 grade 2) and five high-grade HD patients (2 grade 3, plus 3 grade 4). The characteristics of these human samples are shown in Supplementary Table S1. One-way ANOVA followed by Dunnet *post hoc* test showed a significant (***p<0.001) decrease of heteromer expression compared to control subjects. Further details of statistical analyses are given in Supplementary Table S2. (h) Scheme depicting the proposed location and G protein-coupling of the A_2A_R-CB_1_R heteromer in the dorsal striatum. It is currently believed (*left*) that the A_2A_R-CB_1_R heteromer is located on corticostriatal projections as well as on the somatodendritic compartment of indirect-pathway MSNs. Each protomer would maintain its canonical G protein coupling (G_s_ for A_2A_R, and G_i_ for CB_1_R). In this study we propose (*middle*) that the A_2A_R-CB_1_R heteromer is located mostly on indirect-pathway MSNs, not only on their somatodendritic compartment but also likely on their terminals. According to our data, the A_2A_R-CB_1_R heteromer would facilitate G_q_ rather than G_s_ or G_i_ coupling. In symptomatic HD (*right*), the A_2A_R-CB_1_R heteromer would be disrupted into its constituting protomers.

We next investigated whether the aforementioned changes in A_2A_R-CB_1_R heteromer expression found in HD mouse models are also evident in HD. Thus, we used the PLA technique to analyze human caudate-putamen *post mortem* samples from control subjects and HD patients at different grades. A_2A_R-CB_1_R heteromers were readily evident in the caudate-putamen of control individuals, with a high fraction (~65%) of total cells expressing heteromers (Figure 5a and g, and Supplementary Table S1). These complexes were also detected at those normal levels in asymptomatic huntingtin gene-mutation carriers (HD grade 0) and early symptomatic HD patients (HD grades 1–2) (Figure 5b–d and g, and Supplementary Table S1). In contrast, A_2A_R-CB_1_R heteromers were strongly reduced in caudate-putamen samples from high-grade, advanced HD patients (HD grades 3–4), with only ~10% of total cells containing PLA-positive dots (Figure 5e–g, and Supplementary Table S1). PLA labeling was quite uniform in the caudate-putamen sections analyzed, and thus no perceptible differences in A_2A_R-CB_1_R heteromer expression were detected between those two nuclei within each subject (Supplementary Figure S8a and b). In addition, the demographic characteristics of the samples used indicated that the control, low-grade HD and high-grade HD subject populations were rather homogeneous (Supplementary Table S1), thus supporting that the differences found in A_2A_R-CB_1_R heteromer expression were not due to those confounding factors. Taken together, these data support that the human brain expresses A_2A_R-CB_1_R heteromers, and suggest that these complexes might serve specific functions that are impaired at late stages of HD progression.

## DISCUSSION

Despite the progress made toward identifying and understanding GPCR heteromers, their promise as precision drug targets has yet to be fully realized due to the lack of detailed expression maps and functional profiles. A first important conclusion of our study refers to the precise location of the A_2A_R-CB_1_R heteromer in the mouse dorsal striatum. The current view in the field supports that a major site of A_2A_R and CB_1_R colocalization is the corticostriatal-neuron terminal, at which the two receptors could physically interact to form A_2A_R-CB_1_R heteromers (Figure 5h). These presynaptic heteromers have been suggested to provide a frame to explain, at least in part, the negative pharmacological interactions between A_2A_R and CB_1_R that occur in the corticostriatal pathway (Ferre *et al*, 2010; Tebano *et al*, 2012; Ferreira *et al*, 2015; Chiodi *et al*, 2016). However, those previous studies on A_2A_R-CB_1_R heteromers, although elegant and carefully conducted, lacked state-of-the-art genetic controls and heteromer-detecting techniques. Thus, to evaluate the possible existence of A_2A_R-CB_1_R heteromers in corticostriatal neurons, we have made use of three potent genetic models, namely (i) mice lacking CB_1_R selectively in cortical glutamatergic neurons, (ii) CB_1_R-deficient mice in which CB_1_R expression is selectively rescued in cortical glutamatergic neurons, and (iii) CB_1_R-floxed mice in which CB_1_R is selectively excised in corticostriatal neurons. Systematic PLA assays conducted in these mouse models strikingly showed that the expression of the A_2A_R-CB_1_R heteromer in corticostriatal projections to the dorsal striatum is negligible (Figure 1c). This finding supports that the inhibitory cross-talk processes between A_2A_R and CB_1_R reported to date in corticostriatal terminals do not rely primarily on physical interactions between the two receptors at the plasma membrane, but on other potential factors such us an opposite G_s_/G_i_ protein-dependent downstream signaling converging on glutamate release at the presynapse, which, in turn, would conceivably lead to an opposite modulation of the mGluR_5_/phospholipase C-β/diacylglycerol lipase-α (DAGLα)/2-arachidonoylglycerol (2-AG) retrograde-signaling machinery at the postsynapse (Uchigashima *et al*, 2007; Katona and Freund, 2008). In any case, this observed absence of presynaptic A_2A_R-CB_1_R heteromers does certainly not preclude that A_2A_R and CB_1_R could interact with other partners at corticostriatal terminals to form GPCR complexes, for example, the A_1_R-A_2A_R heteromer (Ciruela *et al*, 2006; Quiroz *et al*, 2009).

Another widely accepted site at which striatal A_2A_R-CB_1_R heteromers are believed to reside is the somatodendritic compartment of MSNs, the main target of corticostriatal inputs (Carriba *et al*, 2007; Schiffmann *et al*, 2007; Ferre *et al*, 2010) (Figure 5h). Here, by using (i) mice lacking CB_1_R selectively in GABAergic neurons, (ii) CB_1_R-deficient mice in which CB_1_R expression is selectively rescued in GABAergic neurons, (iii) mice lacking CB_1_R selectively in D_1_R-expressing MSNs, and (iv) CB_1_R-floxed mice in which CB_1_R is selectively excised in MSNs, we cogently demonstrated that the A_2A_R-CB_1_R heteromer is indeed present in indirect-pathway MSNs (Figure 1 and Supplementary Figure S2). It is well established that CB_1_R is largely a presynaptic receptor that is highly abundant in the resident collaterals and long-range projections of MSNs (Uchigashima *et al*, 2007; Katona and Freund, 2008). Our data support that A_2A_R-CB_1_R heteromers are not solely expressed in the somatodendritic compartment of indirect-pathway MSNs, but, most likely, also at terminals of these neurons (Figure 5h). Nonetheless, the higher PLA signal found in GABA-CB_1_R^−/−^ and GABA-Glu-CB_1_R^−/−^mice compared with full CB_1_R^−/−^ mice (Figure 1c and Supplementary Figure S1c) suggests that, in the dorsal striatum, A_2A_R-CB_1_R heteromers may also be located on non-GABAergic, non-glutamatergic cells/terminals such as cholinergic interneurons, dopaminergic projections, or astrocytes. We are also aware that understanding the precise role of A_2A_R-CB_1_R complexes in indirect-pathway MSNs is an extremely complex issue. This complexity is due, in part, to the possibility that A_2A_R and CB_1_R can interact with other receptors in indirect-pathway MSNs. For example, A_2A_R is highly co-expressed with both D_2_R and mGluR_5_, which colocalizes with DAGLα at the perisynaptic border of dendritic spines of MSNs (Uchigashima *et al*, 2007; Katona and Freund, 2008). The activation of mGluR_5_ by glutamate spillover derived from corticostriatal overactivity, which leads to DAGLα-mediated 2-AG generation, can be tuned by D_2_R in MSN dendritic spines (Kreitzer and Malenka, 2005; Yin and Lovinger, 2006). In addition, A_2A_R antagonists potentiate 2-AG release and long-term depression in indirect-pathway MSNs (Lerner *et al*, 2010). Whether these intricate interactions between A_2A_R, D_2_R and mGluR_5_ rely, at least in part, on putative A_2A_R-D_2_R-mGluR_5_ heteromers (Cabello *et al*, 2009) has still to be defined. To complicate the situation further, postsynaptic A_2A_R and D_2_R might form other higher-order heteromeric complexes, including a proposed A_2A_R-CB_1_R-D_2_R heteromer (Navarro *et al*, 2010; Bonaventura *et al*, 2014). This functional conundrum notwithstanding, the present study provides a cogent understanding of the anatomical distribution of the A_2A_R-CB_1_R heteromer, or the complexes containing the heteromer, in the corticostriatal circuit.

Our data also support that the selective coupling to G_q_ protein, rather than to G_s_ or G_i_ proteins, is a biochemical hallmark of the A_2A_R-CB_1_R heteromer in striatal cells (Figure 5h). A G protein switch has in fact been suggested to occur in several GPCR heteromerization processes. For example, a change from the archetypical G_s_-coupled D_1_R (either as monomer or as D_1_R-D_1_R homomers) to non-canonical G_i_-coupled D_1_R-HT_3_R heteromer has been observed (Ferrada *et al*, 2009). In addition, formation of the CB_1_R-5-HT_2A_R heteromer may lead to a switch in G protein coupling for 5-HT_2A_R from G_q_ to G_i_ protein (Viñals *et al*, 2015). Thus, it is possible that in a striatopallidal MSN, there is a coexistence of A_2A_R and CB_1_R (as both monomers and A_2A_R-A_2A_R and CB_1_R-CB_1_R homomers), which are widely believed to couple to G_s/olf_ and G_i_ proteins, respectively, together with A_2A_R-CB_1_R heteromers, which could couple non-canonically to G_q_ protein. How these processes of GPCR protein–protein interaction and subsequent G protein ‘shuffling’ affect corticostriatal circuitry is as yet unknown. It is conceivable that the arrangement of the aforementioned heteromers from A_2A_R and CB_1_R protomers in striatopallidal MSNs, by recruiting activatory G_q_ proteins, would be a way to fuel the indirect pathway and therefore blunt motor activity. However, such a functional outcome is difficult to predict as, according to the currently accepted models of basal ganglia function, motor activation relies on the simultaneous and coordinated activation of the direct and indirect striatal pathways (Nelson and Kreitzer, 2014). In any case, our data support the existence of different pools of A_2A_R and CB_1_R with different G protein coupling in corticostriatal projections, striatopallidal MSNs and striatonigral MSNs, thus providing adenosinergic and cannabinergic cross-signaling with an extreme degree of complexity.

To study whether the A_2A_R-CB_1_R heteromer is affected in a pathological setting we selected HD as the archetypal neurodegenerative disease that primarily affects MSNs in a selective manner. A significant number of studies have dealt with CB_1_R expression and function in HD. In particular, a downregulation of CB_1_R expression has been documented in the caudate-putamen of HD patients and the dorsal striatum of some HD animal models, which seems to reflect the characteristic damage pattern of MSNs (Glass *et al*, 2000; Fernandez-Ruiz *et al*, 2011). In addition, we (Blazquez *et al*, 2011) and others (Mievis *et al*, 2011b) have demonstrated a neuroprotective role of CB_1_R in transgenic mouse models of HD. Likewise, administration of the cannabinoid agonist THC to HD mice prevented disease progression as assessed by behavioral, neuropathological, and molecular markers (Blazquez *et al*, 2011). In sum, it is currently believed that CB_1_R may be neuroprotective in HD. Regarding A_2A_R, its expression has been shown to decrease in striatopallidal MSNs from the caudate-putamen of HD patients and the dorsal striatum of some HD animal models (Glass *et al*, 2000; Lee and Chern, 2014). However, the precise role of A_2A_R in HD progression is not obvious yet, as conflicting results have been reported. Thus, administration of the A_2A_R agonist CGS21680 to HD mice prevented neuropathological deficits and improved motor alterations, although it had no effect on body weight or lifespan (Chou *et al*, 2005). Likewise, the dual-function compound T1–11, which simultaneously activates A_2A_R and blocks adenosine transport, improved motor coordination deficits, reduced striatal huntingtin aggregates, and normalized proteasomal activity (Huang *et al*, 2011). Genetic ablation of A_2A_R in HD mice worsened motor performance, decreased animal survival, and reduced striatal enkephalin expression (Mievis *et al*, 2011a), and also reversed working memory deficits (Li *et al*, 2015). However, and in striking contrast, administration of the A_2A_R antagonist SCH58261 exerted beneficial effects in HD mice by attenuating anxiety-like responses and sensitivity to excitotoxins, although it had no effect on motor coordination (Domenici *et al*, 2007). Because of these (at least apparently) contradictory data coming from various A_2A_R gain-of-function and loss-of-function approaches, it is conceivable that A_2A_R can mediate different (even opposing) molecular and physiopathological mechanisms depending on its cellular location and, hence, its extent of heteromerization. It has been proposed that a selective functional impairment of A_2A_R located on striatopallidal MSNs occurs at pre-symptomatic stages of HD, whereas presynaptic A_2A_R function is not affected (Orru *et al*, 2011). Of note, CB_1_R is also lost in MSNs but not in corticostriatal projections along HD progression (Chiodi *et al*, 2012; Chiarlone *et al*, 2014). This suggests that the corticostriatal pool of non-heteromerizing A_2A_R and CB_1_R would be the main target of adenosinergic and cannabinergic drugs aimed at relieving the symptoms of HD at late stages, whereas the MSN pool of A_2A_R-CB_1_R heteromers could be an additional target of those drugs at early disease stages. As A_2A_R-CB_1_R heteromers are lost in the caudate-putamen of high-grade HD patients, the heteromer’s specific functions would be impaired at advanced stages of HD progression. Thus, the fine negative cross-talk between adenosine and endocannabinoids would conceivably disappear in advanced HD, and one might speculate that the G_q_ specific signaling would be lost as well at those late disease stages (Figure 5h). The A_2A_R-CB_1_R heteromer is singular in both its specific localization on indirect-pathway MSNs and its biochemical characteristics owing to its coupling to non-canonical G_q_-mediated signaling. Together, our findings may open a new conceptual framework to understand the role of coordinated adenosine-cannabinoid function in the indirect striatal pathway, which may be relevant in motor function and neural diseases.

## FUNDING AND DISCLOSURE

This work was supported by grants from the Spanish Ministry of Economy and Competitiveness (MINECO/FEDER; grant SAF2015-64945-R to MG; grant SAF-2014-54840-R to EIC and VC; grant SAF2015-65034-R to PG; grant SAF2015-67474-R to SG; grants SAF2014-55700-P and PCIN-2013-019-C03-03 to FC); Centro de Investigación Biomédica en Red sobre Enfermedades Neurodegenerativas (CIBERNED; grant PI2013/05 to MG, PJM and EIC); Comunidad de Madrid (grant S2010/BMD-2308 to MG); Generalitat de Catalunya (grant 2014-SGR-1236 to EIC); ‘La Marató de TV3’ Foundation (grant 20140610 to EIC; grant 20152031 to FC); Agentschap voor Innovatie door Wetenschap en Technologie (grant SBO-140028 to FC); BBSRC DTP studentship (to PJM and LB); EPSRC (grant EP/M006379/1 to LAH); Deutsche Forschungsgemeinschaft (DFG; grant MO 1920/1-1 to KM; grant CRC-TRR 58 to BL); Institute of Health Carlos III from the Spanish Ministry of Economy and Competitiveness (grant PIE14/00034 to FC; grant PI10/00172 and funding from FEDER grants to MJC and JP); The Basque Government (grant IT764-13 to PG); University of the Basque Country UPV/EHU (grant UFI11/41 to PG); Red de Trastornos Adictivos-Institute of Health Carlos III (grant RD12/0028/0004 to PG). AC is supported by the Spanish Ministry of Economy and Competitiveness (Juan de la Cierva Program). MM is supported by the Spanish Ministry of Education, Culture and Sport (FPU Program). The authors declare no conflict of interest.
