# Supplementary material for: Singular Location and Signaling Profile of Adenosine A2A-Cannabinoid CB1 Receptor Heteromers in the Dorsal Striatum
Source: Neuropsychopharmacology. 2017 Feb 15;43(5):964–77. doi: 10.1038/npp.2017.12 (PMC5854787; doi:10.1038/npp.2017.12)
Supplement: Supplementary Information [file npp201712x1.pdf]

SUPPLEMENTARY INFORMATION

SUPPLEMENTARY MATERIALS AND METHODS

Genetic mouse models to study the location of the A_{2A}R-CB_1R heteromer

We used conditional mutant mice, generated by the Cre/loxP technology, in which the CB_1R gene is primarily absent from forebrain GABAergic neurons ($CB_1^{floxed/floxed;Dlx5/6\text{-}Cre/+}$ mice; herein referred to as GABA-$CB_1^{-/-}$ mice) (Monory *et al*, 2006); from cortical glutamatergic neurons of the dorsal telencephalon ($CB_1^{floxed/floxed;Nex\text{-}Cre/+}$ mice; herein referred to as Glu-$CB_1^{-/-}$ mice) (Monory *et al*, 2006); from both these neuronal populations ($CB_1R^{floxed/floxed;Dlx5/6\text{-}Cre;Nex\text{-}Cre}$ mice; herein referred to as GABA-Glu-$CB_1R^{-/-}$ mice) (Bellocchio *et al*, 2010); from dopamine D_1 receptor (D_1R)-expressing neurons ($CB_1R^{floxed/floxed;Drd1a\text{-}Cre/+}$ mice; herein referred to as D_1R-$CB_1R^{-/-}$ mice) (Monory *et al*, 2007); or from all body cells (full $CB_1R^{-/-}$ mice) (Marsicano *et al*, 2002). We also made use of a Cre-mediated, lineage-specific CB_1R re-expression/rescue strategy in a CB_1R-null background (herein referred to as Stop-CB_1R mice) (Ruehle *et al*, 2013). CB_1R expression was selectively rescued in forebrain GABAergic neurons (herein referred to as GABA-CB_1R-RS mice) by expressing Cre under the regulatory elements of the Dlx5/6 gene (de Salas-Quiroga *et al*, 2015). CB_1R expression was selectively recued in dorsal telencephalic glutamatergic neurons (herein referred to as Glu-CB_1R-RS mice) by expressing Cre under the regulatory elements of the Nex gene (Ruehle *et al*, 2013). As a control, an EIIa-Cre-mediated, global CB_1R expression-rescue in a CB_1R-null background was conducted (herein referred to as CB_1R-RS mice) (Ruehle *et al*, 2013). Full $A_{2A}R^{-/-}$ mice (Ledent *et al*, 1997) were also used. All the experimental procedures used were performed in accordance with the guidelines and with the approval of the Animal Welfare

Committee of Universidad Complutense de Madrid and Comunidad de Madrid, and in accordance with the directives of the European Commission.

Mouse models of Huntington's disease (HD)

Knock-in mice with targeted insertion of 109 CAG repeats that extends the glutamine segment in murine huntingtin to 111 residues, and the corresponding littermates having 7 glutamine residues, were maintained as described (Lloret *et al*, 2006). $Hdh^{Q7/Q111}$ heterozygous males and females were intercrossed to generate age-matched $Hdh^{Q7/Q111}$ heterozygous and $Hdh^{Q7/Q7}$ wild-type littermates. Hemizygous male mice transgenic for exon 1 of the human huntingtin gene with a greatly expanded CAG tract (R6/1 mice, ~115 CAG repeats; R6/2 mice, ~160 CAG repeats) (Mangiarini *et al*, 1996) and wild-type littermates were purchased from The Jackson Laboratory (Bar Harbor, ME, USA). The colonies were maintained as described (Blazquez *et al*, 2011). Animals were housed under a 12 h light/dark cycle with food and water *ad libitum*. All the experimental procedures on HD mice were performed in accordance with the guidelines and with the approval of the Animal Welfare Committees of Universitat de Barcelona and Generalitat de Catalunya, as well as of Universidad Complutense de Madrid, Hospital Ramón y Cajal and Comunidad de Madrid, and in accordance with the directives of the European Commission.

Human brain samples

Paraffin-embedded *post-mortem* 4 μm-thick brain sections containing caudate-putamen were obtained and provided by the Tissue Bank at Hospital Universitario Fundación Alcorcón (Madrid, Spain) and the Netherlands Brain Bank (Amsterdam, The Netherlands) according to the standardized procedures of both institutions. The samples analyzed (4 sections per

individual) were from 10 patients with HD and 5 control subjects with no neurological disease. The characteristics of these samples are given in Supplementary Table S1.

Viral vectors

HA-tagged Cre recombinase, or EGFP as control, was subcloned in a recombinant adeno-associated virus (rAAV) expression vector with a minimal CaMKIIα promoter (kindly provided by Dr. Karl Deisseroth, Stanford University, Stanford, CA, USA) by using standard molecular cloning techniques. All vectors used were of an AAV1/AAV2 mixed serotype, and were generated by calcium phosphate transfection of HEK-293T cells and subsequent purification as described (Monory *et al*, 2006). Eight week-old $CB_1R^{floxed/floxed}$ mice were injected stereotactically with CaMKIIα-Cre-rAAV or CaMKIIα-EGFP-rAAV (in 1.5 µl PBS) either into the dorsal striatum or into the motor cortex projecting onto the dorsal striatum (Chiarlone *et al*, 2014). In the case of the striatum, each animal received one bilateral injection at coordinates (to bregma): antero-posterior +0.6, lateral ±2.0, dorso-ventral -3.0. In the case of the cortex, each animal received 2 bilateral injections at coordinates (to bregma): antero-posterior +1.5, lateral ±1.2, dorso-ventral -1.7; and antero-posterior -0.5, lateral ±1.2, dorso-ventral -1.2. The placement of the rAAV vectors within the dorsal striatum has been previously described (Figure S5A in Chiarlone *et al*, 2014; Figure 4b in Blazquez *et al*, 2015). Six-eight weeks later mice were sacrificed by intracardial perfusion and their brains were excised for PLA analysis.

HIV TAT peptides

Peptides with the sequence of transmembrane domains (TM) of CB_1R fused to HIV transactivator of transcription (TAT) peptide were used as heteromer-disrupting molecules. A cell-penetrating HIV TAT peptide (YGRKKRRQRRR) allows intracellular delivery of fused

peptides (Schwarze *et al*, 1999). This HIV TAT peptide can be inserted effectively into the plasma membrane as a result of both the penetration capacity of the TAT peptide and the hydrophobic property of the TM domain (He *et al*, 2011). To obtain the right orientation of the inserted peptide, the HIV-TAT peptide was fused to the *C*-terminus of TM5 and TM7 or to the *N*-terminus of TM6 of CB_1R (Viñals *et al*, 2015). The sequence of the fusion peptides was the following:

TM5, ETYLMFWIGVTSVLLLFIVYAYMYILW GRKKRRQRRR

TM6, GRKKRRQRRR KTLVLILVVLIICWGPLLAIMVYDVF

TM7, LIKTVFAFCSMLCLLNSTVNPIIYALR GRKKRRQRRR

Cell culture and transfection

Conditionally immortalized striatal neuroblasts obtained from wild-type mice ($STHdh^{Q7/Q7}$ cells) or knock-in mice expressing two copies of a mutant huntingtin allele ($STHdh^{Q111/Q111}$ cells), thus expressing endogenous levels of full-length huntingtin with only 7 glutamines or with 111 glutamines in the protein *N*-terminal domain, respectively, were used (Trettel *et al*, 2000). Cells had been infected with a defective retrovirus transducing the temperature-sensitive A58/U19 large T antigen, and were grown at the permissive temperature of 33ºC in Dulbecco's modified Eagle's medium (DMEM) supplemented with 10% fetal bovine serum, 1 mM sodium pyruvate, 2 mM L-glutamine, 1% streptomycin/penicillin and 400 µg/ml geneticin. HEK-293T cells were grown in DMEM supplemented with 2 mM L-glutamine, 100 µg/ml sodium pyruvate, 100 U/ml penicillin/streptomycin, essential medium non-essential amino acids solution (1/100) and 5% (v/v) heat inactivated fetal bovine serum (all from Invitrogen, Paisley, UK) and were maintained at 37ºC in an atmosphere with 5% CO_2. Cells growing in 6-well dishes were transiently transfected with the corresponding protein cDNA by the polyethylenimine method (Sigma, Steinheim, Germany). Cells were incubated

with the corresponding cDNA together with polyethylenimine (5.47 mM in nitrogen residues) and 150 mM NaCl in a serum-starved medium. After 4 h, the medium was changed to a fresh complete culture medium. Forty-eight h after transfection, cells were washed twice in quick succession in HBSS with 10 mM glucose, detached, and resuspended in the same buffer. To control cell number, sample protein concentration was determined using a Bradford assay kit (Bio-Rad, Munich, Germany).

In Situ Proximity Ligation Assays (PLA)

$STHdh^{Q7/Q7}$ and $STHdh^{Q111/Q111}$ cells were grown on glass coverslips and fixed in 4% paraformaldehyde for 15 min, washed with phosphate-buffered saline (PBS) containing 20 mM glycine, permeabilized with the same buffer containing 0.05% Triton X-100, and successively washed with PBS. Mice were deeply anesthetized and immediately perfused transcardially with PBS followed by 4% paraformaldehyde/phosphate buffer. Brains were removed and post-fixed overnight in the same solution, cryoprotected by immersion in 10, 20, 30% gradient sucrose (24 h for each sucrose gradient) at 4ºC, and then frozen in dry ice-cooled methylbutane. Serial coronal cryostat sections (30 μm-thick) through the whole brain were collected in cryoprotective solution and stored at -20ºC until PLA experiments were performed. Immediately before the assay, mouse brain sections were mounted on glass slides, washed in PBS, permeabilized with PBS containing 0.01% Triton X-100 for 10 min, and successively washed with PBS. Human brain slices were deparaffinized and antigen retrieval was performed with citrate buffer at pH 6.0. In all cases, heteromers were detected using the Duolink II in situ PLA detection Kit (OLink; Bioscience, Uppsala, Sweden) following supplier's instructions. A mixture of the primary antibodies [mouse anti-$A_{2A}R$ antibody (1:100; Millipore, Darmstadt, Germany; cat #05-717) and rabbit anti-CB_1R antibody (1:100; Thermo Scientific, Fremont, CA, USA; cat #PA1-745)] was routinely used to detect $A_{2A}R$-

CB_1R heteromers together with PLA probes detecting mouse or rabbit antibodies (Supplementary Figure S1a-c). The specificity of these antibodies for PLA assays has been previously reported by both us (Bonaventura *et al*, 2015; Viñals *et al*, 2015) and others (Trifilieff *et al*, 2011; Sierra *et al*, 2015). In addition, PLA experiments conducted with a different anti-CB_1R primary antibody (1:100; Frontier Institute, Japan; cat #CB1-Rb-Af380) provided a similar $A_{2A}R$-CB_1R heteromer detection in mouse dorsal-striatum preparations (Supplementary Figure S1d). Moreover, the specificity of the three aforementioned primary antibodies was also demonstrated by immunocytofluorescence studies, conducted under comparable conditions as the PLA assays, in HEK-293T cells transfected or not with cDNAs encoding human $A_{2A}R$ or human CB_1R (Supplementary Figure S1e). To detect CB_1R-D_2R heteromers, the anti-D_2R antibody (1:100; Millipore; cat #AB5084P) was directly coupled to a plus DNA chain and the anti-CB_1 antibody was directly coupled to a minus DNA chain following supplier's instructions. Then, samples were processed for ligation and amplification with a Detection Reagent Red and were mounted using a DAPI-containing mounting medium. Samples were analyzed in a Leica SP2 confocal microscope (Leica Microsystems, Mannheim, Germany) equipped with an apochromatic 63X oil-immersion objective (1.4 numerical aperture), and a 405 nm and a 561 nm laser lines. Three serial coronal sections (30-µm thick) per animal spaced 0.24 mm apart containing the dorsal striatum were used. For each field of view a stack of two channels (one per staining) and 9 to 15 Z-stacks with a step size of 1 µm were acquired. Images were opened and processed with Image J software (National Institutes of Health, Bethesda, MD). Quantification of cells containing one or more red dots versus total cells (blue nuclei) was determined by using the Fiji package (http://pacific.mpi-cbg.de). In some experiments, the total number of red dots found in stained cells versus total cells (blue nuclei) was determined. Nuclei and red dots were counted on the maximum projections of each image stack. After getting the projection, each channel was processed individually. The

blue nuclei and red dots were segmented by filtering with a median filter, subtracting the background, enhancing the contrast with the Contrast Limited Adaptive Histogram Equalization (CLAHE) plug-in, and finally applying a threshold to obtain the binary image and the regions of interest (ROIs). When indicated, after image processing, the red channel was depicted in green color to facilitate detection on blue stained nuclei, always maintaining the color intensity settings constant for all images. Sample analysis and data acquisition were conducted in a blinded manner.

Fluorescence complementation assays

To obtain plasmids for fusion proteins expression, sequences encoding amino acid residues 1-155 and 156-238 of YFP Venus protein were subcloned in the pcDNA3.1 vector to obtain the YFP Venus hemi-truncated proteins (nVenus and cVenus). The cDNAs for human $A_{2A}R$ and D_1R were amplified without their stop codons using sense and antisense primers harboring unique EcoRI and BamHI sites. The cDNA for human CB_1R was amplified without their stop codons using sense and antisense primers harboring unique EcoRI and KpnI. The amplified fragments were subcloned to be in-frame with restriction sites of pcDNA3.1-cVenus or pcDNA3.1-nVenus vectors to give the plasmids that express proteins fused to hemi-YFP Venus on the C-terminal end ($A_{2A}R$-cYFP, D_1R-cYFP or CB_1R-nYFP). For fluorescence complementation assays, HEK-293T cells were transiently co-transfected with the cDNA encoding receptor fused to nYFP and receptor fused to cYFP. After 48 h, cells were treated or not with the indicated TM-TAT peptides (4 μM) for 4 h at 37 °C. To quantify protein-reconstituted YFP Venus expression, cells (20 μg protein) were distributed in 96-well microplates (black plates with a transparent bottom, Porvair, King's Lynn, UK), and emission fluorescence at 530 nm was monitored in a FLUOstar Optima Fluorimeter (BMG Lab Technologies, Offenburg, Germany) equipped with a high-energy xenon flash lamp, using a

10 nm bandwidth excitation filter at 400 nm reading. Protein fluorescence expression was determined as the fluorescence of the sample minus the fluorescence of cells not expressing the fusion proteins (basal). Cells expressing $A_{2A}R$-cVenus and nVenus or CB_1R-nVenus and cVenus showed similar fluorescence levels than non-transfected cells.

Dynamic Mass Redistribution (DMR) assays

The cell signaling signature was determined using an EnSpire® Multimode Plate Reader (PerkinElmer, Waltham, MA, USA) by a label-free technology. Refractive waveguide grating optical biosensors, integrated in 384-well microplates, allow extremely sensitive measurements of changes in local optical density in a detecting zone up to 150 nm above the surface of the sensor. Cellular mass movements induced upon receptor activation were detected by illuminating the underside of the biosensor with polychromatic light and measured as changes in wavelength of the reflected monochromatic light that is a sensitive function of the index of refraction. The magnitude of this wavelength shift (in picometers) is directly proportional to the amount of DMR. Briefly, 24 h before the assay, cells were seeded at a density of 10,000-12,000 cells per well in 384-well sensor microplates with 30 µl growth medium and cultured for 24 h (37°C, 5% CO_2) to obtain 70-80% confluent monolayers. Previous to the assay, cells were washed twice with assay buffer (HBSS with 20 mM Hepes, pH 7.15) and incubated for 2 h in 30 µl per well of assay-buffer with 0.1% DMSO in the reader at 24°C. Hereafter, the sensor plate was scanned and a baseline optical signature was recorded before adding 10 µl of test compound dissolved in assay buffer containing 0.1% DMSO. Then, DMR responses were monitored for at least 5,000 s. Kinetic results were analyzed using EnSpire Workstation Software v 4.10.

Determination of cAMP concentration

Homogeneous time-resolved fluorescence energy transfer (HTRF) assays were performed using the Lance Ultra cAMP kit (PerkinElmer), based on competitive displacement of a europium chelate-labelled cAMP tracer bound to a specific antibody conjugated to acceptor beads. We first established the optimal cell density for an appropriate fluorescent signal. This was done by measuring the TR-FRET signal determined as a function of forskolin concentration using different cell densities. The forskolin dose-response curves were related to the cAMP standard curve in order to establish which cell density provides a response that covers most of the dynamic range of the cAMP standard curve. Cells (500-1000 per well) growing in medium containing 50 µM zardeverine were pre-treated with toxins or the corresponding vehicle in white ProxiPlate 384-well microplates (PerkinElmer) at 25°C for the indicated time, and stimulated with agonists for 15 min before adding 0.5 µM forskolin or vehicle, and incubating for an additional 15 min period. Fluorescence at 665 nm was analyzed on a PHERAstar Flagship microplate reader equipped with an HTRF optical module (BMG Lab technologies, Offenburg, Germany).

Determination of Ca^{2+} concentration

To determine cytosolic Ca^{2+} free concentration, striatal cells were transfected with 4 µg of GCaMP6 calcium sensor (Chen *et al*, 2013) using Lipofectamine 3000. After 48 h, cells were incubated (0.2 mg of protein/ml in 96-well black, clear bottom microtiter plates) with Mg^{2+}-free Locke's buffer (in mM: 154 NaCl, 5.6 KCl, 3.6 $NaHCO_3$, 2.3 $CaCl_2$, 5.6 glucose and 5 HEPES, pH 7.4) supplemented with 10 µM glycine. After the addition of receptor ligands at the indicated concentrations, fluorescence emission intensity of GCaMP6 was recorded at 515 nm upon excitation at 488 nm on an EnSpire® Multimode Plate Reader (PerkinElmer) for 255 s every 15 s and 100 flashes per well.

Determination of ERK and Akt phosphorylation

Mouse brains were rapidly removed and placed in ice-cold oxygenated (95% O_2 and 5% CO_2) Krebs-HCO_3^- buffer containing (in mM): 124 NaCl, 4 KCl, 1.25 KH_2PO_4, 1.5 $MgCl_2$, 1.5 $CaCl_2$, 10 glucose and 26 $NaHCO_3$, pH 7.4. The brains were sliced coronally at 4°C in ice-cold buffer, and slices (500-μm thick) were transferred into an incubation tube containing 1 ml of buffer. The temperature was raised to 23°C and, after 30 min, the medium was replaced by 2 ml of buffer (23°C), containing or not 4 μM TM-TAT peptide, and incubated under constant oxygenation (95% O_2 and 5% CO_2) at 30°C for 4-5 h in an Eppendorf Thermomixer (5 Prime, Inc., Boulder, CO, USA). The media was replaced by 200 μl of buffer and incubated for 30 min before the addition of any agent. Slices were treated or not with the indicated ligand for the indicated times, and then frozen in dry ice and stored at -80 °C until use. Slices were rinsed with ice-cold PBS and lysed by the addition of 500 μl of ice-cold lysis buffer (50 mM Tris-HCl, pH 7.4, 50 mM NaF, 150 mM NaCl, 45 mM glycerophosphate, 1% Triton X- 100, 20 μM phenyl-arsine oxide, 0.4 mM $NaVO_4$, and protease inhibitor mixture). The cellular debris was removed by centrifugation at 13,000 g for 5 min at 4°C, and the protein was quantified by the bicinchoninic acid method using bovine serum albumin dilutions as standard. Equivalent amounts of protein (10 μg) were separated by electrophoresis on a denaturing 10% SDS-polyacrylamide gel and transferred onto PVDF-fluorescence membranes. Odyssey blocking buffer (LI-COR Biosciences) was then added, and the membrane was rocked for 90 min. Membranes were routinely probed for 2-3 h with a mixture of a mouse anti-phospho-ERK1/2 antibody (1:2500; Sigma; cat #M8159), a rabbit anti-phospho-Ser473-Akt antibody (1:2500; SAB Signalway Antibody, Pearland, USA; cat #11054), and a rabbit anti-ERK1/2 antibody that recognizes both phosphorylated and non-phosphorylated ERK1/2 (1:40,000; Sigma; cat #M5670). Alternatively, when indicated, Akt activation was determined by probing membranes with a mixture of the aforementioned rabbit

anti-phospho-Akt and anti-total-ERK antibodies, plus a mouse anti-total-Akt antibody (1:2500; Cell Signaling, Danvers, MA, USA; cat #2920). Bands were visualized by the addition of a mixture of IRDye 800 (anti-mouse) antibody (1:10,000; Sigma) and IRDye 680 (anti-rabbit) antibody (1:10,000; Sigma) for 1 h and scanned by the Odyssey infrared scanner (LI-COR Biosciences). Band densities were quantified at exposure times within the dynamic range (not saturated, not overexposed) using the scanner software exported to Excel (Microsoft). Due to the high sensitivity of the anti-total-ERK antibody, the levels of phosphorylated ERK1/2 or phosphorylated Akt were routinely normalized for differences in loading using the total ERK protein band optical density. The anti-total-ERK antibody works better in our hands than the anti-total-Akt antibody. In any event, we have not detected any significant variability with using anti-total-ERK, anti-total-Akt or anti-actin loading controls with respect to the pERK or pAkt signals in these short-term cell signaling experiments.

Western blot of CB_1R and $A_{2A}R$

Brain tissue from wild-type $Hdh^{Q7/Q7}$ and heterozygous mutant $Hdh^{Q7/Q111}$ mice at 6 months of age was homogenized in ice-cold lysis buffer [20 mM Tris, pH 8.0, 150 mM NaCl, 50 mM NaF, 1% NP-40 and 10% glycerol, supplemented with 1 mM sodium orthovanadate and protease inhibitor cocktail (Sigma)], cleared by centrifugation at 16,000 g for 20 min, and the supernatants collected. Following determination of protein concentration, extracts were mixed with 5× SDS sample buffer, resolved on 8% SDS–PAGE and transferred to nitrocellulose membranes (Whatman Schleicher & Schuell, Keene, NH, USA). After incubation (30 min) in blocking buffer containing 10% non-fat powdered milk in Tris buffered saline-Tween (TBS-T), membranes were blotted overnight at 4°C with anti-$A_{2A}R$ (1:500; Santa Cruz Biotechnology; cat #sc-32261) and CB_1R (1:1000; Frontier Institute; cat #CB1-Rb-Af380) primary antibodies. Membranes were then rinsed 3 times with TBS-T and incubated with

horseradish peroxidase-conjugated secondary antibody for 1 h at room temperature. After washing for 30 min with TBS-T, membranes were developed using an enhanced chemiluminescence ECL kit (Santa Cruz Biotechnology). The Gel-Pro densitometry program (Gel-Pro Analyzer for Windows, version 4.0.00.001) was used to quantify the different immunoreactive bands relative to the intensity of the actin band (loading control) in the same membranes within a linear range of detection for the ECL reagent.

Determination of $A_{2A}R$ and CB_1R immunoreactivity

Wild-type $Hdh^{Q7/Q7}$ and heterozygous mutant $Hdh^{Q7/Q111}$ mice at 6 months of age were perfused transcardially with PBS followed by 4% paraformaldehyde/phosphate buffer. Brains were removed and postfixed overnight, cryoprotected, and frozen in dry ice-cooled methylbutane. Serial coronal cryostat sections (30 μm-thick) through the whole brain were collected in PBS as free-floating sections and stored at -20ºC. Immunofluorescence staining was performed as previously described (Puigdellivol *et al*, 2015). Briefly, sections were rinsed three times in PBS and permeabilized and blocked in PBS containing 0.3% Triton X-100 and 3% donkey serum (Pierce Biotechnology, Rockford, IL, USA) for 15 min at room temperature. The sections were then washed in PBS and incubated overnight at 4°C with rabbit anti-CB_1R (1:500; Frontier Institute; cat #CB1-Rb-Af380) or goat anti-$A_{2A}R$ (1:200; Frontier Institute; cat #A2A-Go-Af700) antibodies, that were detected with Cy3 anti-rabbit or FITC anti-goat secondary antibodies (1:200; Jackson ImmunoResearch, West Grove, PA, USA), respectively. For $A_{2A}R$ staining, an antigen retrieval protocol was performed before immunofluorescence. Brain slices were incubated with sodium citrate buffer (10 mM sodium citrate, 0.05% Tween 20, pH 6.0) for 30 min at 90ºC in a pre-heated water bath and then removed to room temperature for 20 min. Before permeabilization and blocking, the slices were washed 3 times in PBS. Images were acquired with Zeiss LSM510 META confocal

microscope with argon and HeNe lasers. Images were taken using a 63X numerical aperture objective with 1X digital zoom and standard (one Airy disc) pinhole. Three serial coronal sections (30-µm thick) per animal spaced 0.24 mm apart containing the dorsal striatum were used. For each slice, we obtained 3 fields per dorsal striatum region. In each field, an entire Z-stack was obtained, and optical sections (between 3 and 5 per field) of 0.5 µm were collected separately (4 µm) in order to avoid biased counting. CB_1R and $A_{2A}R$ immunoreactivity were quantified using ImageJ software. None of the secondary antibodies produced any signal in preparations incubated in the absence of the corresponding primary antibodies. Sample analysis and data acquisition were conducted in a blinded manner.

Statistics

Unless otherwise indicated, data are presented as mean ± SEM. Statistical analyses were conducted with unpaired Student's t test or with one-way ANOVA, followed by Bonferroni or Dunnett *post hoc* test, as appropriate. Details of the statistical analyses conducted for each set of data are given in Supplementary Table S2. A p value of less than 0.05 was considered significant.

References for Supplementary Materials and Methods

Bellocchio L, Lafenetre P, Cannich A, Cota D, Puente N, Grandes P *et al* (2010). Bimodal control of stimulated food intake by the endocannabinoid system. *Nat Neurosci* **13:** 281-283.

Blazquez C, Chiarlone A, Bellocchio L, Resel E, Pruunsild P, Garcia-Rincon D *et al* (2015). The CB_1 cannabinoid receptor signals striatal neuroprotection via a PI3K/Akt/mTORC1/BDNF pathway. *Cell Death Differ* **22:** 1618-1629.

Blazquez C, Chiarlone A, Sagredo O, Aguado T, Pazos MR, Resel E *et al* (2011). Loss of striatal type 1 cannabinoid receptors is a key pathogenic factor in Huntington's disease. *Brain* **134:** 119-136.

Bonaventura J, Navarro G, Casado-Anguera V, Azdad K, Rea W, Moreno E *et al* (2015). Allosteric interactions between agonists and antagonists within the adenosine A_{2A} receptor-dopamine D_2 receptor heterotetramer. *Proc Natl Acad Sci USA* **112:** E3609-3618.

Chen TW, Wardill TJ, Sun Y, Pulver SR, Renninger SL, Baohan A *et al* (2013). Ultrasensitive fluorescent proteins for imaging neuronal activity. *Nature* **499:** 295-300.

Chiarlone A, Bellocchio L, Blazquez C, Resel E, Soria-Gomez E, Cannich A *et al* (2014). A restricted population of CB_1 cannabinoid receptors with neuroprotective activity. *Proc Natl Acad Sci USA* **111:** 8257-8262.

De Salas-Quiroga A, Diaz-Alonso J, Garcia-Rincon D, Remmers F, Vega D, Gomez-Canas M *et al* (2015). Prenatal exposure to cannabinoids evokes long-lasting functional alterations by targeting CB_1 receptors on developing cortical neurons. *Proc Natl Acad Sci USA* **112:** 13693-13698.

He SQ, Zhang ZN, Guan JS, Liu HR, Zhao B, Wang HB *et al* (2011). Facilitation of mu-opioid receptor activity by preventing delta-opioid receptor-mediated codegradation. *Neuron* **69:** 120-131.

Ledent C, Vaugeois JM, Schiffmann SN, Pedrazzini T, El Yacoubi M, Vanderhaeghen JJ *et al* (1997). Aggressiveness, hypoalgesia and high blood pressure in mice lacking the adenosine A2a receptor. *Nature* **388:** 674-678.

Lloret A, Dragileva E, Teed A, Espinola J, Fossale E, Gillis T *et al* (2006). Genetic background modifies nuclear mutant huntingtin accumulation and HD CAG repeat instability in Huntington's disease knock-in mice. *Hum Mol Genet* **15:** 2015-2024.

Mangiarini L, Sathasivam K, Seller M, Cozens B, Harper A, Hetherington C *et al* (1996). Exon 1 of the HD gene with an expanded CAG repeat is sufficient to cause a progressive neurological phenotype in transgenic mice. *Cell* **87:** 493-506.

Marsicano G, Wotjak CT, Azad SC, Bisogno T, Rammes G, Cascio MG *et al* (2002). The endogenous cannabinoid system controls extinction of aversive memories. *Nature* **418:** 530-534.

Monory K, Blaudzun H, Massa F, Kaiser N, Lemberger T, Schutz G *et al* (2007). Genetic dissection of behavioural and autonomic effects of delta-9-tetrahydrocannabinol in mice. *PLoS Biol* **5:** e269.

Monory K, Massa F, Egertova M, Eder M, Blaudzun H, Westenbroek R *et al* (2006). The endocannabinoid system controls key epileptogenic circuits in the hippocampus. *Neuron* **51:** 455-466.

Puigdellivol M, Cherubini M, Brito V, Giralt A, Suelves N, Ballesteros J *et al* (2015). A role for Kalirin-7 in corticostriatal synaptic dysfunction in Huntington's disease. *Hum Mol Genet* **24:** 7265-7285.

Ruehle S, Remmers F, Romo-Parra H, Massa F, Wickert M, Wortge S *et al* (2013). Cannabinoid CB_1 receptor in dorsal telencephalic glutamatergic neurons: distinctive sufficiency for hippocampus-dependent and amygdala-dependent synaptic and behavioral functions. *J Neurosci* **33:** 10264-10277.

Schwarze SR, Ho A, Vocero-Akbani A, Dowdy SF (1999). In vivo protein transduction: delivery of a biologically active protein into the mouse. *Science* **285:** 1569-1572.

Sierra S, Luquin N, Rico AJ, Gomez-Bautista V, Roda E, Dopeso-Reyes IG *et al* (2015). Detection of cannabinoid receptors CB_1 and CB_2 within basal ganglia output neurons in macaques: changes following experimental parkinsonism. *Brain Struct Funct* **220:** 2721-2738.

Trettel F, Rigamonti D, Hilditch-Maguire P, Wheeler VC, Sharp AH, Persichetti F *et al* (2000). Dominant phenotypes produced by the HD mutation in $STHdh^{Q111}$ striatal cells. *Hum Mol Genet* **9:** 2799-2809.

Trifilieff P, Rives ML, Urizar E, Piskorowski RA, Vishwasrao HD, Castrillon J *et al* (2011). Detection of antigen interactions ex vivo by proximity ligation assay: endogenous dopamine D_2-adenosine A_{2A} receptor complexes in the striatum. *Biotechniques* **51:** 111-118.

Viñals X, Moreno E, Lanfumey L, Cordomi A, Pastor A, de La Torre R *et al* (2015). Cognitive impairment induced by delta-9-tetrahydrocannabinol occurs through heteromers between cannabinoid CB_1 and serotonin 5-HT_{2A} receptors. *PLoS Biol* **13:** e1002194.

Supplementary Table S1. Characteristics of the human samples used.

Subject	Diagnosis	Age	Sex	Sample	PMI (h)	HD medication	Cause of death	PLA
#1	Control	45	M	CP	12	Not applicable	Mors subita	67 ± 8
#2	Control	62	F	CP	8	Not applicable	Mors subita	72 ± 11
#3	Control	35	M	CP	24	Not applicable	Mors subita	67 ± 2
#4	Control	74	M	CP	≤ 24	Not applicable	Mors subita	55 ± 2
#5	Control	79	M	CP	≤ 24	Not applicable	Mors subita	61 ± 5
#6	HD grade 0	67	F	CP	6	Nortryptiline, antidepressants	Euthanasia	61 ± 2
#7	HD grade 1	54	M	P	4	n.a.	Mors subita	70 ± 1
#8	HD grade 1	75	F	CP	≤ 24	n.a.	Mors subita	53 ± 5
#9	HD grade 2	81	M	CP	≤ 24	n.a.	HD	70 ± 1.8
#10	HD grade 2	69	M	CP	≤ 24	n.a.	HD	59 ± 3.5
#11	HD grade 3	51	M	CP	≤ 24	n.a.	Respiratory infection	4.3 ± 1.4
#12	HD grade 3	51	F	CP	≤ 24	n.a.	HD	0.3 ± 0.3
#13	HD grade 4	47	F	CP	12	Tetrabenazine	Respiratory infection	29 ± 16
#14	HD grade 4	45	M	CP	≤ 24	Tetrabenazine	HD	21 ± 2
#15	HD grade 4	44	M	CP	≤ 24	Pimozide, clonazepam, loprazolam, clorazepam, risperidone	Respiratory infection	21 ± 2

HD, Huntington's disease; M, male; F, female; C, caudate; P, putamen; PMI, post-mortem interval; n.a., non-available information; PLA, proximity ligation assay (values are mean ± SEM of the number of cells containing one or more $A_{2A}R$-CB_1R-positive dots expressed as the percentage of the total number of cell nuclei).

Supplementary Table S2. Statistical details of each set of data.

Figure	Conditions	n*	Analysis (*post-hoc*)	Factor	F value	t value	p value
1c	$CB_1R^{f/f}$ vs. GABA-$CB_1R^{-/-}$, Glu-$CB_1R^{-/-}$, GABA-Glu-$CB_1R^{-/-}$	3	One-way ANOVA (Dunnett)	Genotype	F(3,19) = 7.50	-	< 0.01
1c	CB_1R-RS vs. Stop-CB_1R, GABA-CB_1R-RS, Glu-CB_1R-RS	3	One-way ANOVA (Dunnett)	Genotype	F(3,34) = 30.25	-	< 0.001
1c	$CB_1R^{f/f}$ vs. CB_1R-RS	3	Unpaired Student's t test	Genotype	-	t(12) = 2.113	> 0.05
2a	Basal vs. treatments (pERK)	12	One-way ANOVA (Dunnett)	Treatment	F(9,38) = 4.30	-	< 0.001
2a	Agonist alone vs. agonist plus antagonist (pERK)	12	One-way ANOVA (Bonferroni)	Treatment	F(9,38) = 4.30	-	< 0.001
2a	Basal vs. treatments (pAkt)	12	One-way ANOVA (Dunnett)	Treatment	F(9,35) = 7.43	-	< 0.001
2a	Agonist alone vs. agonist plus antagonist (pAkt)	12	One-way ANOVA (Bonferroni)	Treatment	F(9,35) = 7.43	-	< 0.001
2b	Non-transfected cells vs. transfected cells	3	One-way ANOVA (Dunnett)	Transfection	F(8,43) = 125.3	-	< 0.001
2b	No peptide vs. peptide	3	One-way ANOVA (Bonferroni)	Treatment	F(8,43) = 125.3	-	< 0.001
2c	Basal vs. treatments (pERK, TM5)	12	One-way ANOVA (Dunnett)	Treatment	F(5,12) = 7.28	-	< 0.01
2c	Agonist alone vs. agonist plus antagonist (pERK, TM5)	12	One-way ANOVA (Bonferroni)	Treatment	F(3,8) = 1.259	-	> 0.05
2c	Basal vs. treatments (pERK, TM6)	12	One-way ANOVA (Dunnett)	Treatment	F(5,12) = 4.571	-	< 0.05
2c	Agonist alone vs. agonist plus antagonist (pERK, TM6)	12	One-way ANOVA (Bonferroni)	Treatment	F(5,12) = 4.571	-	> 0.05
2c	Basal vs. treatments (pERK, TM5+TM6)	12	One-way ANOVA (Dunnett)	Treatment	F(5,12) = 5.289	-	< 0.05
2c	Agonist alone vs. agonist plus antagonist (pERK, TM5+TM6)	12	One-way ANOVA (Bonferroni)	Treatment	F(3,8) = 0.2214	-	> 0.05
2c	Basal vs. treatments (pERK, TM7)	12	One-way ANOVA (Dunnett)	Treatment	F(5,16) = 4.709	-	< 0.01
2c	Agonist alone vs. agonist plus antagonist (pERK, TM7)	12	One-way ANOVA (Bonferroni)	Treatment	F(5,16) = 4.709	-	< 0.01
2c	Basal vs. treatments (pAkt, TM5)	12	One-way ANOVA (Dunnett)	Treatment	F(5,18) = 2.236	-	< 0.05
2c	Agonist alone vs. agonist plus antagonist (pAkt, TM5)	12	One-way ANOVA (Bonferroni)	Treatment	F(5,18) = 2.236	-	> 0.05
2c	Basal vs. treatments (pAkt, TM6)	12	One-way ANOVA (Dunnett)	Treatment	F(5,16) = 2.285	-	< 0.05
2c	Agonist alone vs. agonist plus antagonist (pAkt, TM6)	12	One-way ANOVA (Bonferroni)	Treatment	F(5,16) = 2.285	-	> 0.05
2c	Basal vs. treatments (pAkt, TM5+TM6)	12	One-way ANOVA (Dunnett)	Treatment	F(5,13) = 7.634	-	< 0.01
2c	Agonist alone vs. agonist plus antagonist (pAkt, TM5+TM6)	12	One-way ANOVA (Bonferroni)	Treatment	F(3,9) = 0.0795	-	> 0.05
2c	Basal vs. treatments (pAkt, TM7)	12	One-way ANOVA (Dunnett)	Treatment	F(5,14) = 3.579	-	< 0.05
2c	Agonist alone vs. agonist plus antagonist (pAkt, TM7)	12	One-way ANOVA (Bonferroni)	Treatment	F(4,12) = 5.561	-	< 0.01
4a	$Hdh^{Q7/Q111}$ vs. age-matched $Hdh^{Q7/Q7}$	5	One-way ANOVA (Bonferroni)	Genotype	F(5,91) = 32.00	-	< 0.01
4b	Basal vs. treatments	5-6	One-way ANOVA (Dunnett)	Treatment	F(5,18) = 14.94	-	< 0.001
4b	Agonist alone vs. agonist plus antagonist	5-6	One-way ANOVA (Bonferroni)	Treatment	F(5,18) = 14.94	-	< 0.001
4c	Basal vs. treatments	5-6	One-way ANOVA (Dunnett)	Treatment	F(5,28) = 4.96	-	< 0.01
4c	Agonist alone vs. agonist plus antagonist	5-6	One-way ANOVA (Bonferroni)	Treatment	F(5,28) = 4.96	-	< 0.01

4d	Basal vs. treatments	5-6	One-way ANOVA (Dunnett)	Treatment	F(5,30) = 24.03	-	< 0.001
4d	Agonist alone vs. agonist plus antagonist	5-6	One-way ANOVA (Bonferroni)	Treatment	F(3,20) = 0.3274	-	> 0.05
4e	Basal vs. treatments	5-6	One-way ANOVA (Dunnett)	Treatment	F(5,19) = 7.131	-	< 0.001
4e	Agonist alone vs. agonist plus antagonist	5-6	One-way ANOVA (Bonferroni)	Treatment	F(3,14) = 0.0389	-	> 0.05
5g	Control vs. low-grade HD, high-grade HD	5	One-way ANOVA (Dunnett)	Group	F(2,84) = 68.75	-	< 0.001
S1c	$CB_1R^{+/+}$ vs. $CB_1R^{+/+}$ only $A_{2A}R$ Ab, $CB_1R^{+/+}$ only CB_1R Ab, Full $CB_1R^{-/-}$	3	One-way ANOVA (Dunnett)	Genotype	F(3,15) = 47.81	-	< 0.001
S1c	$A_{2A}R^{+/+}$ vs. $A_{2A}R^{+/+}$ only $A_{2A}R$ Ab, $A_{2A}R^{+/+}$ only CB_1R Ab, Full $A_{2A}R^{-/-}$	3	One-way ANOVA (Dunnett)	Genotype	F(3,30) = 35.47	-	< 0.001
S1c	$CB_1R^{+/+}$ vs. $A_{2A}R^{+/+}$	3	Unpaired Student's t test	Genotype	-	t(14) = 1.864	> 0.05
S4a	Basal vs. treatments ($STHdh^{Q7}$)	3	One-way ANOVA (Dunnett)	Treatment	F(11,24) = 114.1	-	< 0.001
S4a	Basal vs. treatments ($STHdh^{Q7}$ + PTX)	3	One-way ANOVA (Dunnett)	Treatment	F(11,24) = 160.7	-	< 0.001
S4a	Basal vs. treatments ($STHdh^{Q7}$ + CTX)	3	One-way ANOVA (Dunnett)	Treatment	F(11,24) = 18.11	-	< 0.001
S4b	Basal vs. treatments ($STHdh^{Q111}$)	3	One-way ANOVA (Dunnett)	Treatment	F(11,24) = 56.32	-	< 0.001
S4b	Basal vs. treatments ($STHdh^{Q111}$ + PTX)	3	One-way ANOVA (Dunnett)	Treatment	F(11,24) = 27.39	-	< 0.001
S4b	Basal vs. treatments ($STHdh^{Q111}$ + CTX)	3	One-way ANOVA (Dunnett)	Treatment	F(11,24) = 12.05	-	< 0.001
S4c	Basal vs. treatments ($STHdh^{Q7}$, TM5+TM6)	3	One-way ANOVA (Dunnett)	Treatment	F(6,14) = 24.20	-	< 0.001
S4c	Basal vs. treatments ($STHdh^{Q111}$, TM5+TM6)	3	One-way ANOVA (Dunnett)	Treatment	F(6,14) = 49.42	-	< 0.001
S6a	$Hdh^{Q7/Q111}$ vs. $Hdh^{Q7/Q7}$ ($A_{2A}R$)	8-9	Unpaired Student's t test	Genotype	-	t(15) = 3.070	< 0.01
S6a	$Hdh^{Q7/Q111}$ vs. $Hdh^{Q7/Q7}$ (CB_1R)	5	Unpaired Student's t test	Genotype	-	t(8) = 0.5822	> 0.05
S6b	$Hdh^{Q7/Q111}$ vs. $Hdh^{Q7/Q7}$ ($A_{2A}R$)	3	Unpaired Student's t test	Genotype	-	t(52) = 1.557	> 0.05
S6b	$Hdh^{Q7/Q111}$ vs. $Hdh^{Q7/Q7}$ (CB_1R)	3	Unpaired Student's t test	Genotype	-	t(52) = 3.388	< 0.01
S6c	$Hdh^{Q7/Q111}$ vs. $Hdh^{Q7/Q7}$	3	Unpaired Student's t test	Genotype	-	t(17) = 0.5288	> 0.05
S7a	R6/1 vs. age-matched WT	3	One-way ANOVA (Bonferroni)	Genotype	F(9,60) = 6.231	-	< 0.001
S7b	R6/2 vs. WT	3	Unpaired Student's t test	Genotype	-	t(4) = 7.383	< 0.01
S7c	R6/1 vs. WT	3	Unpaired Student's t test	Genotype	-	t(6) = 0.5163	> 0.05
S7c	R6/2 vs. WT	3	Unpaired Student's t test	Genotype	-	t(5) = 3.362	< 0.05

*Number of subjects (human data), number of mice (*ex vivo* animal experiments) or number of independent experiments (*in vitro* cell cultures).

SUPPLEMENTARY FIGURE LEGENDS

Supplementary Figure S1. Controls for the PLA assays of the $A_{2A}R$-CB_1R heteromer. (a-d) PLA assays were performed in dorsal-striatum sections from 3-4 month-old mice of different genotypes. $A_{2A}R$-CB_1R heteromers are shown as green dots. Nuclei are colored in blue by DAPI staining. (a) Representative pictures from $CB_1R^{+/+}$ control mice, from these control mice in the absence of anti-CB_1R primary antibody ($CB_1R^{+/+}$ only $A_{2A}R$ Ab) or anti-$A_{2A}R$ primary antibody ($CB_1R^{+/+}$ only CB_1R Ab), and from full $CB_1R^{-/-}$ mice. (b) Representative pictures from $A_{2A}R^{+/+}$ control mice, from these control mice in the absence of anti-CB_1R primary antibody ($A_{2A}R^{+/+}$ only $A_{2A}R$ Ab) or anti-$A_{2A}R$ primary antibody ($A_{2A}R^{+/+}$ only CB_1R Ab), and from full $A_{2A}R^{-/-}$ mice. Scale bar: 20 µm. (c) Quantification of the number of cells containing one or more dots expressed as the percentage of the total number of cells (blue nuclei). Data are the mean ± SEM of counts in 4-10 different fields from 3 different animals of each type. One-way ANOVA followed by Dunnet *post hoc* test showed a significant (***$p < 0.001$) decrease of heteromer expression compared to the respective $CB_1R^{+/+}$ or $A_{2A}R^{+/+}$ controls. Further details of statistical analyses are given in Supplementary Table S2. (d) Representative pictures from $CB_1R^{+/+}$ mice and full $CB_1R^{-/-}$ mice from PLA assays conducted with a different anti-CB_1R primary antibody (Frontier Institute, Japan; cat #CB1-Rb-Af380). Scale bar: 20 µm. (e) Immunocytofluorescence experiments with anti-$A_{2A}R$ primary antibody (1:100; Millipore, Darmstadt, Germany; cat #05-717; upper panels), anti-CB_1R primary antibody (1:100; Thermo Scientific, Fremont, CA, USA; cat #PA1-745; middle panels) or anti-CB_1R primary antibody (1:100; Frontier Institute, Japan; cat #CB1-Rb-Af380; lower panels), and goat anti-mouse Alexa Fluor 488 (1:300; Invitrogen, Paisley, UK; cat #A-11001) or goat anti-rabbit Alexa Fluor 488 (1:300; Invitrogen; cat #A-11008) as secondary antibodies, in HEK-293T cells transfected or not with cDNAs encoding human

$A_{2A}R$ or human CB_1R. $A_{2A}R$ or CB_1R is labeled in green. Nuclei are colored in blue by DAPI staining. Representative pictures are shown. Scale bar: 20 µm.

Supplementary Figure S2. $A_{2A}R$-CB_1R heteromers are located on indirect-pathway MSNs in the mouse dorsal striatum. PLA assays were performed in brain sections from 3-4 month-old mice of different genotypes without (a) or with (b, c) different rAAV vector injections. $A_{2A}R$-CB_1R heteromers are shown as green dots (a) or red dots (b, c). Nuclei are colored in blue by DAPI staining. (a) Representative pictures of dorsal-striatum sections from D_1R-$CB_1R^{-/-}$ mice and their control $CB_1R^{floxed/floxed}$ ($CB_1R^{f/f}$) mice. Scale bar: 20 µm. (b) $CB_1R^{f/f}$ mice were injected sterotactically with a rAAV vector encoding CaMKIIα-Cre or CaMKIIα-EGFP into the dorsal striatum. Representative pictures from the dorsal striatum and the globus pallidus are shown. Scale bar: 20 µm. (c) $CB_1R^{f/f}$ mice were injected sterotactically with a rAAV vector encoding CaMKIIα-Cre or CaMKIIα-EGFP into the motor cortex. Representative pictures from the motor cortex and the dorsal striatum are shown. Scale bar: 20 µm.

Supplementary Figure S3. Effect of CB_1R agonists on dynamic mass redistribution assays in wild-type $STHdh^{Q7}$ and mutant huntingtin-expressing $STHdh^{Q111}$ striatal neuroblasts. Dynamic mass redistribution (DMR) assays were performed in $STHdh^{Q7}$ (black bars) and $STHdh^{Q111}$ (white bars) cells incubated for 30 min with vehicle and further treated with the CB_1R agonist WIN-55,212-2 (a) or the CB_1R agonist CP-55,940 (b) at the indicated doses. Values are mean ± SEM (n = 3 experiments for each condition) and are expressed as maximal shift of reflected light wavelength (pm) over basal obtained from the corresponding DMR curves (see Figure 3).

Supplementary Figure S4. $A_{2A}R$-CB_1R heteromers do not mediate cAMP signaling in wild-type STHdh^{Q7} and mutant huntingtin-expressing STHdh^{Q111} striatal neuroblasts. In (a, b), cAMP concentration was determined in STHdh^{Q7} (a) and STHdh^{Q111} (b) cells incubated overnight with vehicle or pertussis toxin (PTX; 10 ng/ml), or for 2 h with cholera toxin (CTX; 100 ng/ml), and further treated with vehicle, the $A_{2A}R$ agonist CGS21680 (0.5 μM), the CB_1R agonist WIN-55,212-2 (0.2 μM) or the CB_1R agonist CP-55,940 (0.2 μM), in the absence or presence of 0.5 μM forskolin (FK). In (c), cells were pretreated for 4 h with medium or with 4 μM TM5 plus TM6 before the addition of the drugs as indicated above. Values are mean ± SEM (n = 3 experiments for each condition) and are expressed as percentage of forskolin-induced cAMP accumulation (100%, dotted line). One-way ANOVA followed by Dunnet *post hoc* test showed a significant (*p < 0.05, **p < 0.01, ***p < 0.01) difference over basal. Further details of statistical analyses are given in Supplementary Table S2.

Supplementary Figure S5. $A_{2A}R$ and CB_1R agonists increase intracellular free Ca^{2+} concentration in wild-type STHdh^{Q7} and mutant huntingtin-expressing STHdh^{Q111} striatal neuroblasts. Intracellular free Ca^{2+} concentration over time was monitored fluorimetrically in STHdh^{Q7} and STHdh^{Q111} cells treated with vehicle or the $A_{2A}R$ agonist CGS21680 (1 μM), the CB_1R agonist CP-55,940 (1 μM) or the CB_1R agonist WIN-55,212-2 (1 μM). Each panel is a representative experiment of n = 3 different experiments. Each curve is the mean of triplicate determinations (basal, dotted line).

Supplementary Figure S6. The loss of $A_{2A}R$-CB_1R heteromers in Hdh$^{Q7/Q111}$ HD mice at advanced disease stages is not largely due to the mere loss of their constituting protomers. (a) $A_{2A}R$ and CB_1R total protein expression was determined by Western blot in the striatum of 6 month-old wild-type Hdh$^{Q7/Q7}$ and mutant huntingtin-expressing knock-in

Hdh$^{Q7/Q111}$ mice. Data are the mean ± SEM of 5-9 different animals of each type. Representative blots from 3 animals of each genotype are shown. Unpaired Student's t test showed a significant (**$p < 0.01$) decrease of $A_{2A}R$ expression in Hdh$^{Q7/Q111}$compared to the respective age-matched Hdh$^{Q7/Q7}$ mice. (b) $A_{2A}R$ and CB_1R total immunoreactivity was determined by confocal microscopy analysis in the dorsal striatum of 6 month-old Hdh$^{Q7/Q7}$ and Hdh$^{Q7/Q111}$ mice. Data are the mean ± SEM of 3 different animals of each type. Representative pictures are shown. Scale bar: 10 μm. Unpaired Student's t test showed a significant (**$p < 0.01$) decrease of CB_1R expression in Hdh$^{Q7/Q111}$compared to the respective age-matched Hdh$^{Q7/Q7}$ mice. (c) PLA assays were performed in dorsal-striatum sections from 6 month-old Hdh$^{Q7/Q7}$ and Hdh$^{Q7/Q111}$ mice. CB_1R-D_2R heteromers are shown as green dots. Nuclei are colored in blue by DAPI staining. Representative pictures are shown. Scale bar: 20 μm. Quantification of the number of cells containing one or more dots expressed as the percentage of the total number of cells (blue nuclei). Data are the mean ± SEM of counts in 8-11 different fields from 3 different animals of each type. Further details of statistical analyses are shown in Supplementary Table S2.

Supplementary Figure S7. $A_{2A}R$-CB_1R heteromers are expressed in R6 HD mice at early but not advanced disease stages. PLA assays were performed in dorsal-striatum sections from age-matched wild-type mice and mouse models of HD transgenic for human mutant huntingtin exon 1 (R6/1 and R6/2 mice) at symptomatic stages. In (a, b), $A_{2A}R$-CB_1R heteromers are shown as green dots in wild-type and R6/1 mice at 1, 2, 3 and 4 months of age (a) or R6/2 mice at 8 weeks of age (b). In (c), CB_1R-D_2R heteromers are shown as green dots in 4 month-old R6/1 mice or 8 week-old R6/2 mice as well as age-matched wild-type controls. Nuclei are colored in blue by DAPI staining. Representative pictures are shown. Scale bar: 20 μm. In (a-c), graphs show the quantification of the number of cells containing

one or more dots expressed as the percentage of the total number of cells (blue nuclei). Data are the mean ± SEM of counts in 3-14 different fields from 3 different animals of each type. One-way ANOVA followed by Bonferroni *post hoc* test (a), or unpaired Student's t test (b, c), showed a significant (*p < 0.05, **p < 0.01, ***p < 0.001) difference of heteromer expression in R6/1 or R6/2 mice compared to the respective age-matched wild-type mice. Further details of statistical analyses are given in Supplementary Table S2.

Supplementary Figure S8. A_{2A}R-CB_1R heteromer expression in the caudate and the putamen of a representative control subject and a representative high-grade HD patient. PLA assays performed in caudate-putamen sections of *post-mortem* samples from a representative control subject (a; Subject #5 in Supplementary Table S1) and a representative grade 3 HD patient (b; Subject #11 in Supplementary Table S1). A_{2A}R-CB_1R heteromers are shown as green dots. Nuclei are colored in blue by DAPI staining. Values are mean ± SEM of the number of cells containing one or more A_{2A}R-CB_1R-positive dots expressed as the percentage of the total number of cell nuclei. Scale bar: 20 μm.

Figure S1

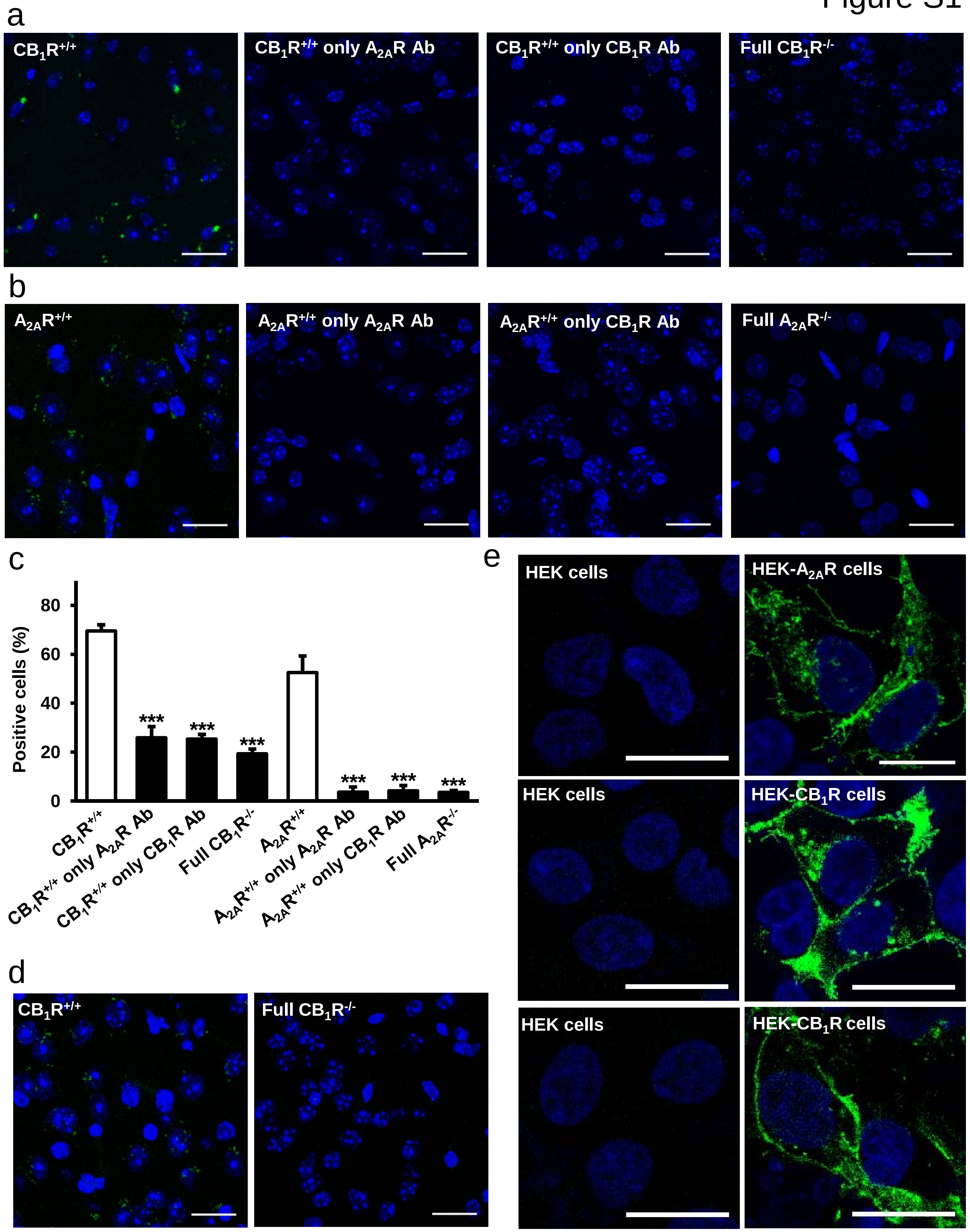
a
CB1R+/+
CB1R+/+ only A2AR Ab
CB1R+/+ only CB1R Ab
Full CB1R-/-
b
A2AR+/+
A2AR+/+ only A2AR Ab
A2AR+/+ only CB1R Ab
Full A2AR-/-
c
Positive cells (%)
80
60
40
20
0
***
***
***
***
***
***
CB1R+/+
CB1R+/+ only A2AR Ab
CB1R+/+ only CB1R Ab
Full CB1R-/-
A2AR+/+
A2AR+/+ only A2AR Ab
A2AR+/+ only CB1R Ab
Full A2AR-/-
d
CB1R+/+
Full CB1R-/-
e
HEK cells
HEK-A2AR cells
HEK cells
HEK-CB1R cells
HEK cells
HEK-CB1R cells

Figure S2

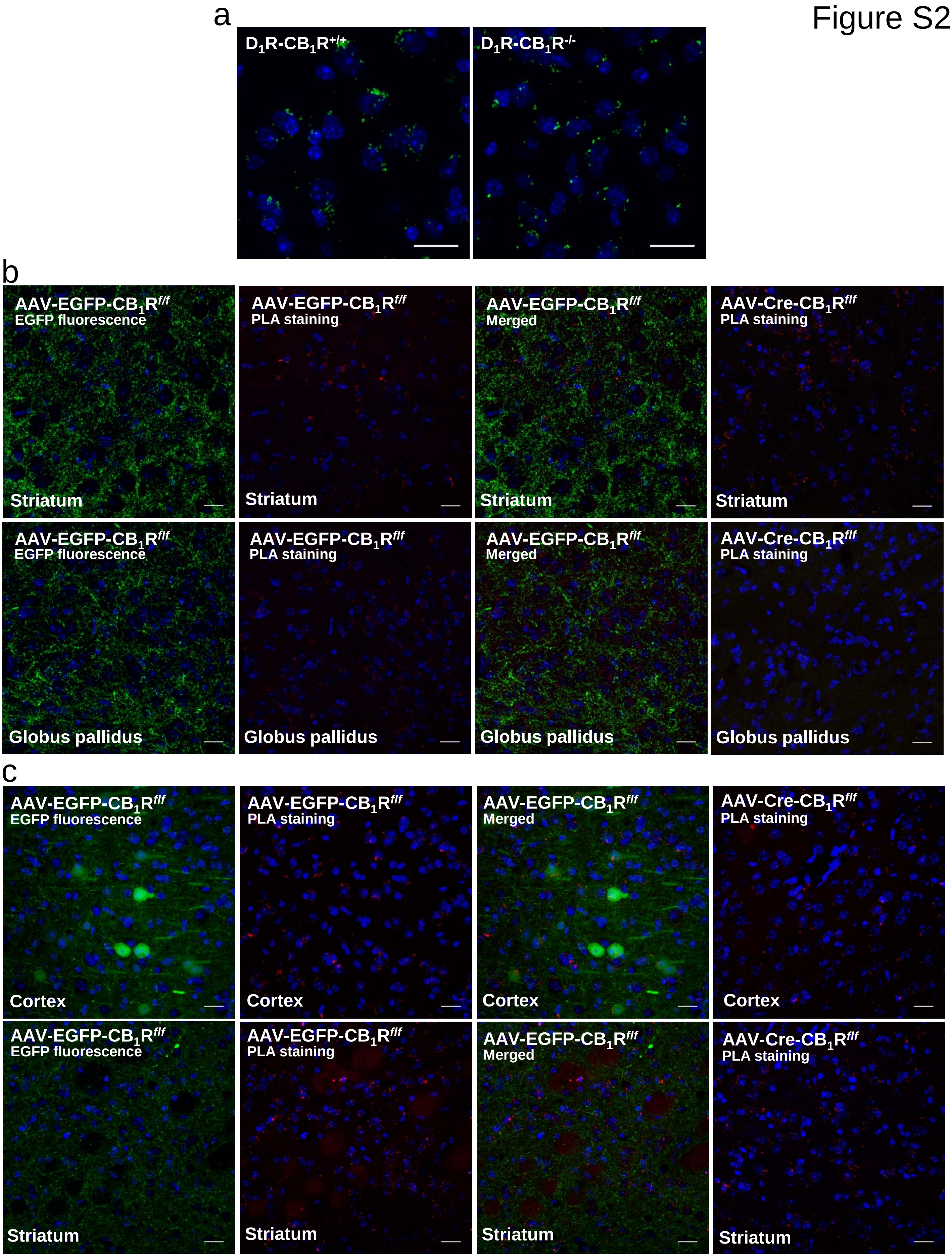
a
D1R-CB1R+/+
D1R-CB1R-/-
b
AAV-EGFP-CB1Rf/f
EGFP fluorescence
Striatum
AAV-EGFP-CB1Rf/f
PLA staining
Striatum
AAV-EGFP-CB1Rf/f
Merged
Striatum
AAV-Cre-CB1Rf/f
PLA staining
Striatum
AAV-EGFP-CB1Rf/f
EGFP fluorescence
Globus pallidus
AAV-EGFP-CB1Rf/f
PLA staining
Globus pallidus
AAV-EGFP-CB1Rf/f
Merged
Globus pallidus
AAV-Cre-CB1Rf/f
PLA staining
Globus pallidus
c
AAV-EGFP-CB1Rf/f
EGFP fluorescence
Cortex
AAV-EGFP-CB1Rf/f
PLA staining
Cortex
AAV-EGFP-CB1Rf/f
Merged
Cortex
AAV-Cre-CB1Rf/f
PLA staining
Cortex
AAV-EGFP-CB1Rf/f
EGFP fluorescence
Striatum
AAV-EGFP-CB1Rf/f
PLA staining
Striatum
AAV-EGFP-CB1Rf/f
Merged
Striatum
AAV-Cre-CB1Rf/f
PLA staining
Striatum

Figure S3

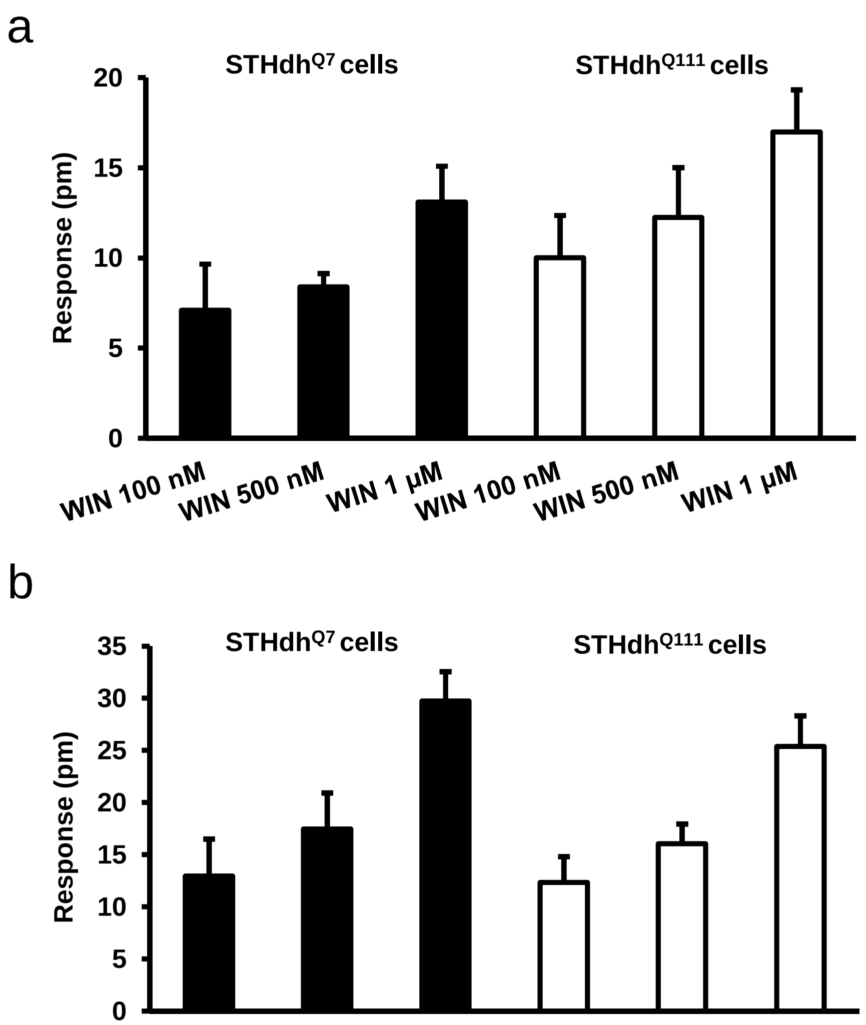
a
STHdhQ7 cells
STHdhQ111 cells
Response (pm)
20
15
10
5
0
WIN 100 nM
WIN 500 nM
WIN 1 µM
WIN 100 nM
WIN 500 nM
WIN 1 µM
b
STHdhQ7 cells
STHdhQ111 cells
Response (pm)
35
30
25
20
15
10
5
0
CP 100 nM
CP 500 nM
CP 1 µM
CP 100 nM
CP 500 nM
CP 1 µM

Figure S4

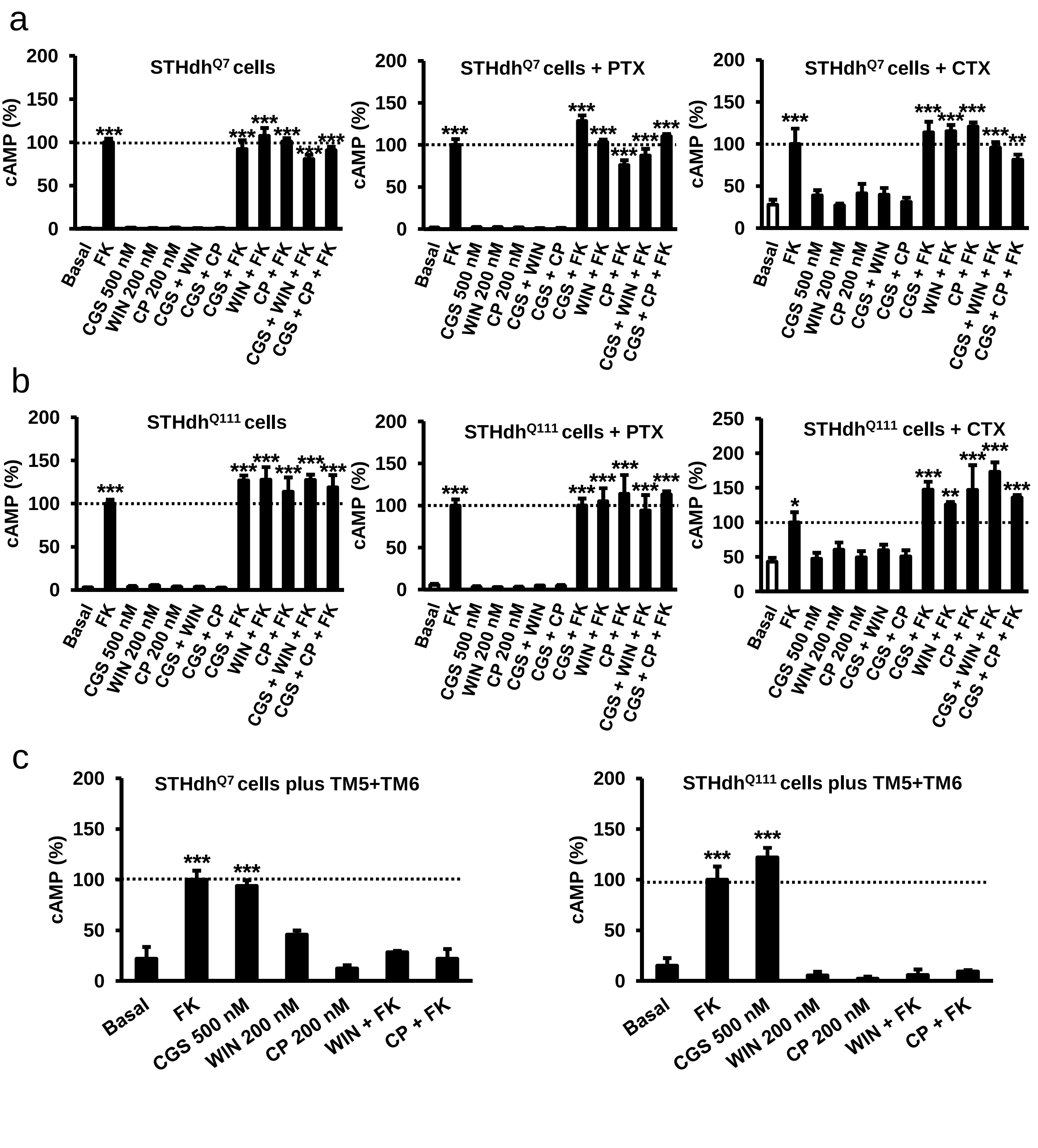
a
STHdhQ7 cells
STHdhQ7 cells + PTX
STHdhQ7 cells + CTX
b
STHdhQ111 cells
STHdhQ111 cells + PTX
STHdhQ111 cells + CTX
c
STHdhQ7 cells plus TM5+TM6
STHdhQ111 cells plus TM5+TM6
cAMP (%)
Basal
FK
CGS 500 nM
WIN 200 nM
CP 200 nM
CGS + WIN
CGS + CP
CGS + FK
WIN + FK
CP + FK
CGS + WIN + FK
CGS + CP + FK

Figure S5

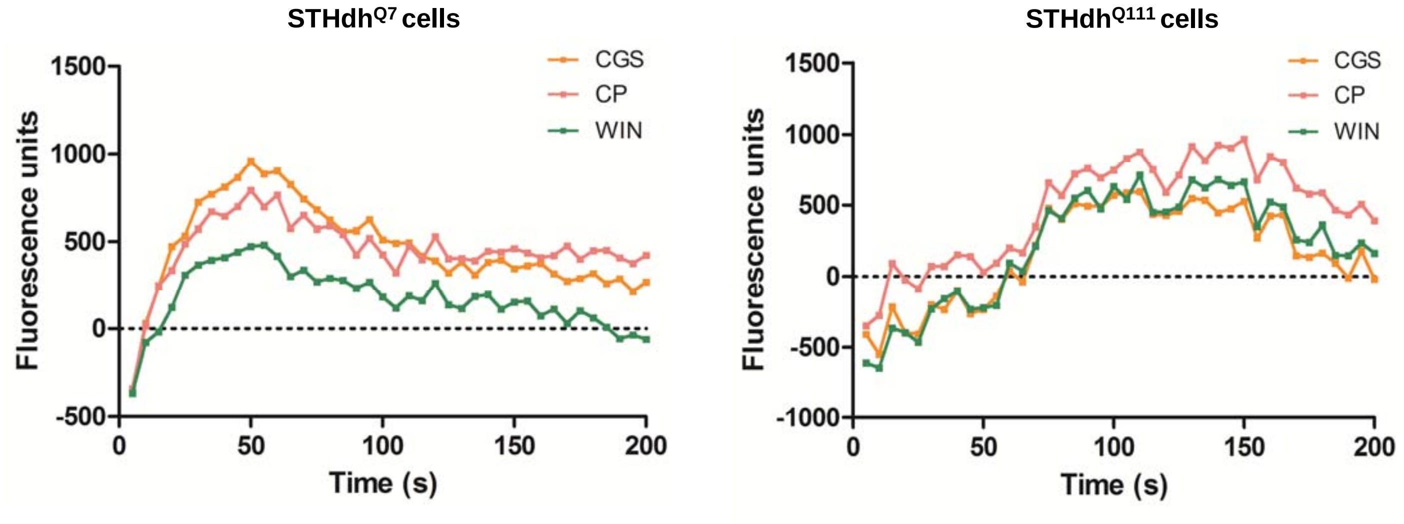
STHdh^{Q7} cells
STHdh^{Q111} cells
CGS
CP
WIN
Fluorescence units
Time (s)
1500
1000
500
0
-500
-1000
0
50
100
150
200

Figure S6

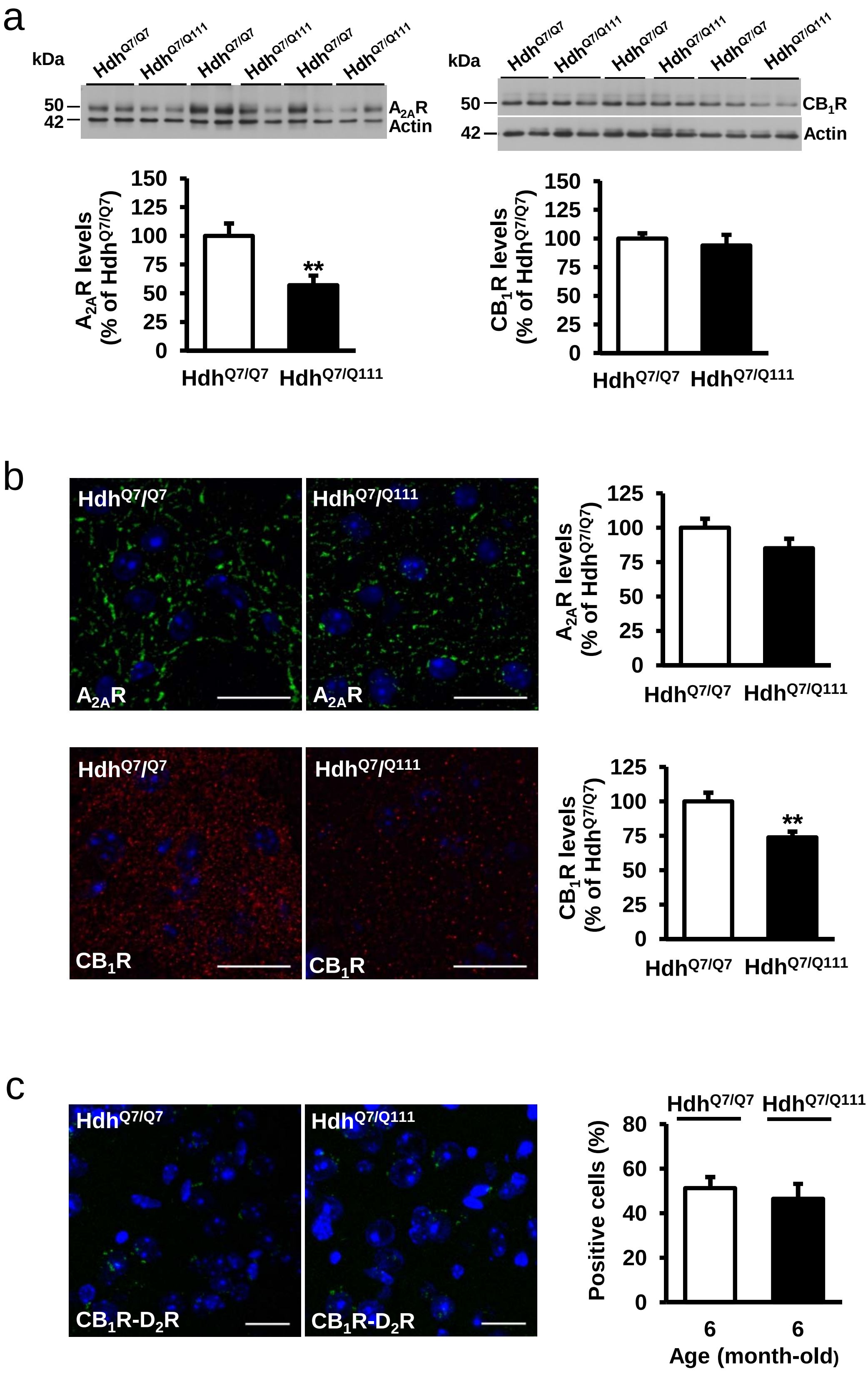

a
kDa
Hdh^Q7/Q7
Hdh^Q7/Q111
50
42
A2AR
Actin
CB1R
150
125
100
75
50
25
0
**
A2AR levels (% of Hdh^Q7/Q7)
CB1R levels (% of Hdh^Q7/Q7)
b
A2AR
CB1R
c
CB1R-D2R
Positive cells (%)
80
60
40
20
6
Age (month-old)

Figure S7

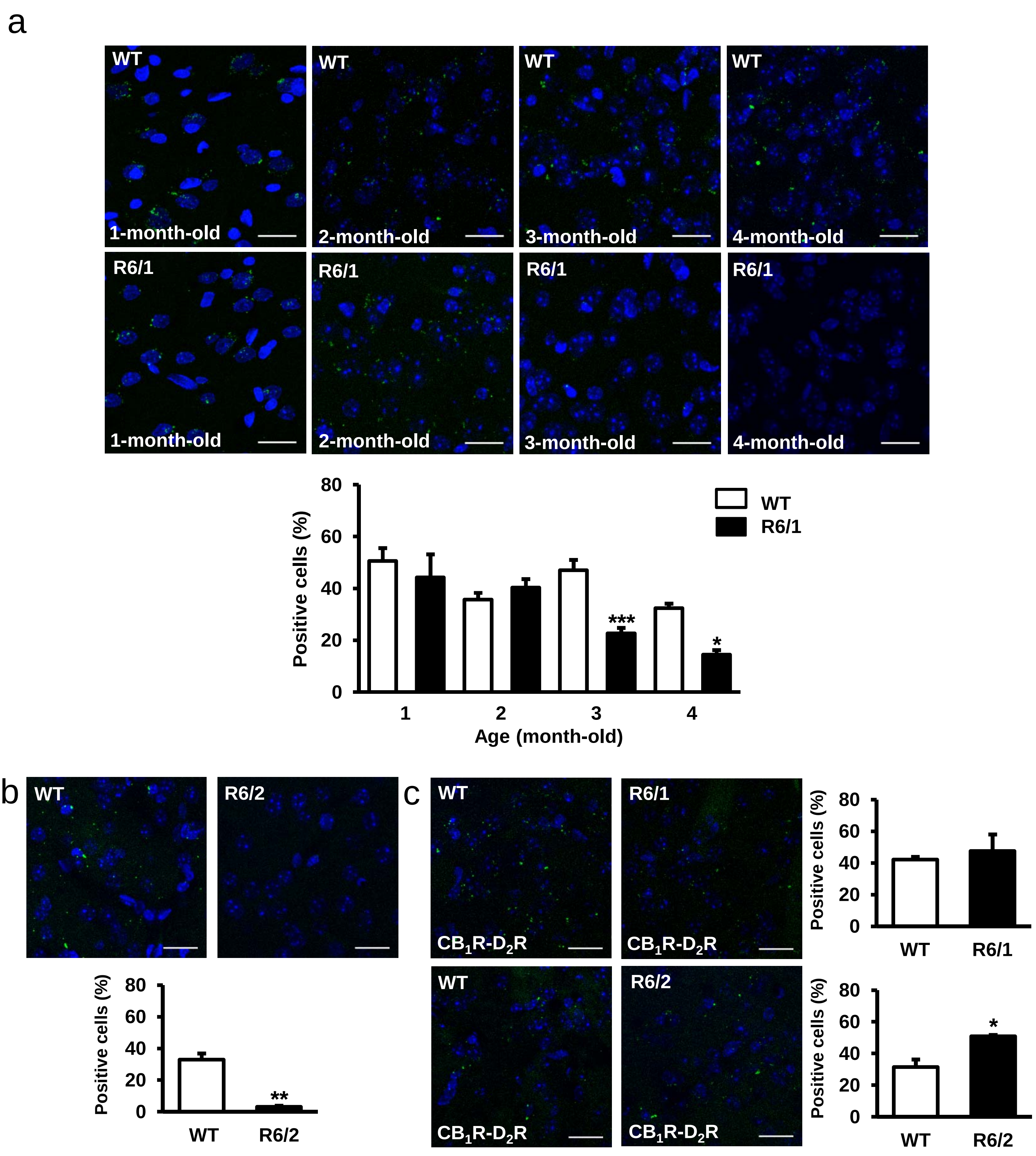
a
WT
1-month-old
WT
2-month-old
WT
3-month-old
WT
4-month-old
R6/1
1-month-old
R6/1
2-month-old
R6/1
3-month-old
R6/1
4-month-old
Positive cells (%)
80
60
40
20
0
***
*
1
2
3
4
Age (month-old)
WT
R6/1
b
WT
R6/2
Positive cells (%)
80
60
40
20
0
**
WT
R6/2
c
WT
R6/1
CB1R-D2R
CB1R-D2R
WT
R6/2
CB1R-D2R
CB1R-D2R
Positive cells (%)
80
60
40
20
0
WT
R6/1
Positive cells (%)
80
60
40
20
0
*
WT
R6/2

Figure S8

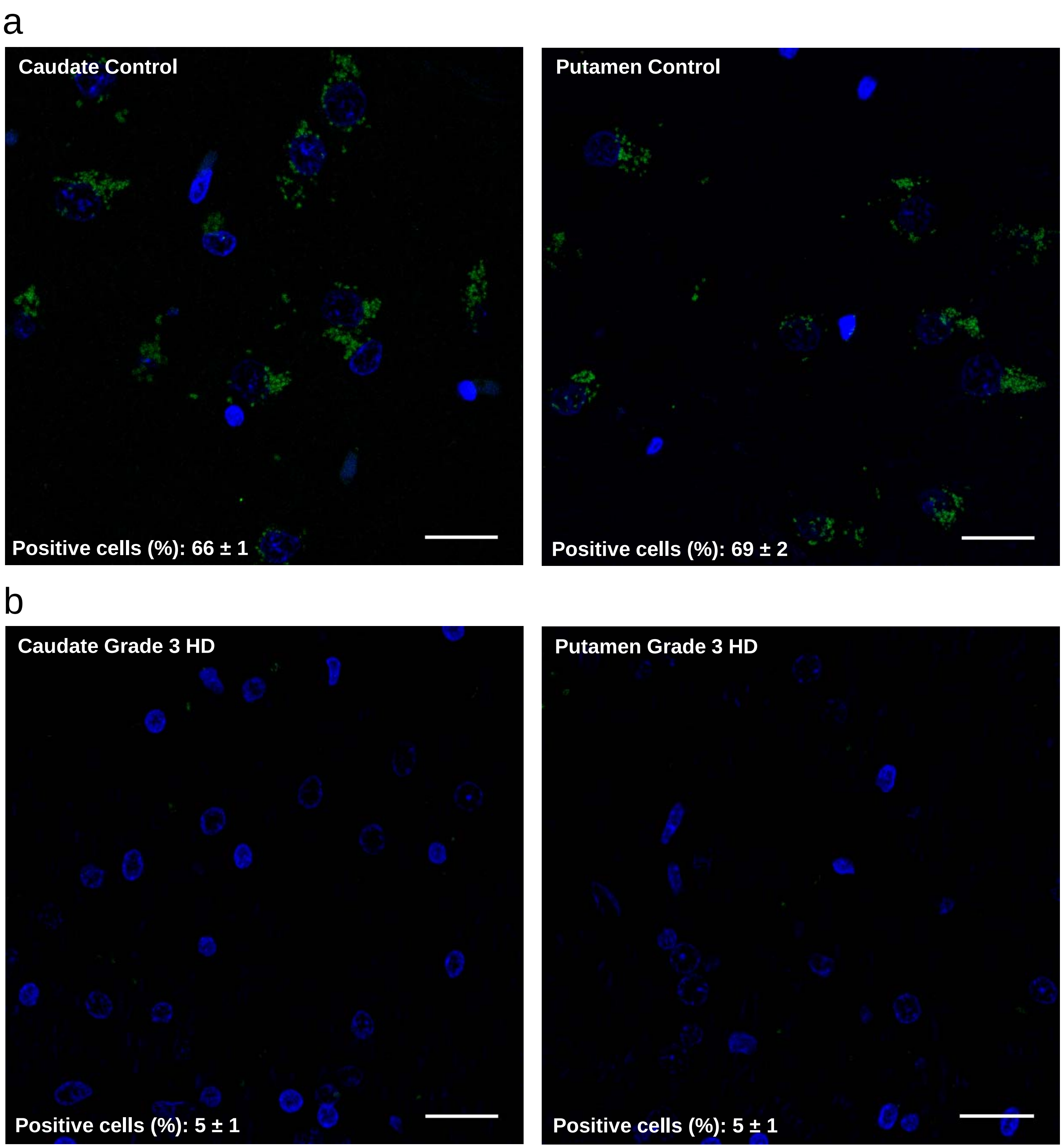
a
Caudate Control
Positive cells (%): 66 ± 1
Putamen Control
Positive cells (%): 69 ± 2
b
Caudate Grade 3 HD
Positive cells (%): 5 ± 1
Putamen Grade 3 HD
Positive cells (%): 5 ± 1
